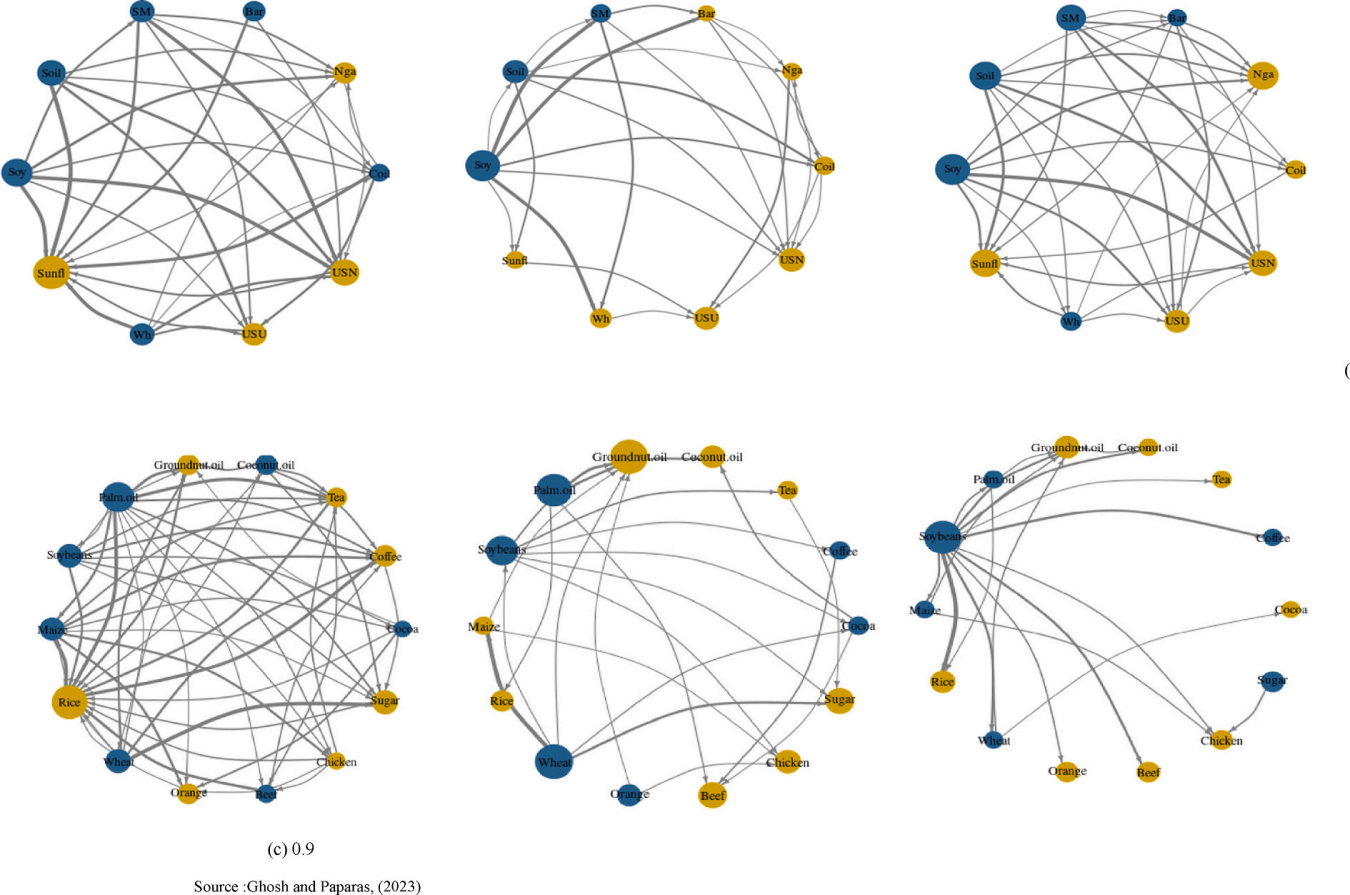# The rise of Soybean in international commodity markets: A quantile investigation

**DOI:** 10.1016/j.heliyon.2024.e34669

**Published:** 2024-07-26

**Authors:** Gustavo María Barboza Martignone, Bikramaditya Ghosh, Dimitrios Papadas, Karl Behrendt

**Affiliations:** a Food Land & Agribusiness Management Department, Harper Adams University, Newport, TF10 8NB, UK; b Symbiosis Institute of Business Management, Symbiosis International, Deemed University, Bengaluru, 560100, India; c AI Deep Economics, Department of Applied Economics, Uruguay

**Keywords:** Commodity markets, Connectedness index, Quantile VAR, Risk spillover, Soybean international market

## Abstract

The complex interplay between agricultural and energy commodities has been a subject of interest in past research, gaining more relevance recently due to geopolitical events such as the conflict between Ukraine and Russia. This conflict has systematically driven up the prices of both energy and agricultural commodities. Deeply understanding the dynamic interconnections between these commodities and the cascading events resulting from the war is crucial for comprehensive market analysis. Our study leverages the connectedness or risk of spillover based on a Quantile Vector Autoregression (QVAR) model, allowing us to track connectedness over time through the examination of extreme quantiles. This approach facilitates the identification of shocks triggered by exogenous events, such as the Russian-Ukrainian war, which are often observable in these extreme quantiles or tails. The investigation encompasses several agricultural commodities: Wheat, Barley, Soybean, Soybean Oil, Soybean Meal, and Sunflower Oil, along with energy commodities represented by Crude Oil and Natural Gas. Furthermore, we considered the prices of crucial fertilizers, DAP & Urea, given their significance in agricultural production. The timeframe for our study extends from January 2010 to January 2023, providing a comprehensive review of market trends during various geopolitical scenarios. This research contributes valuable insights into the intersection of global events, agricultural trends, and energy commodity markets. The study revealed that Soybean and its derivatives consistently play a leading role in the market, with Soybean being the primary shock transmitter. This is particularly true for the upper Quantile, where Soybean and Soybean Meal's influence remains stable. On the other hand, Soybean Oil's, Barley, and Wheat risk of spillover has increased, especially during the Ukraine-Russia conflict. Finally, spillover appears symmetric, with both extreme tails exhibiting around 91–87 % connectedness, while the median Quantile is under 49 %. We observed a diminution in network complexity, manifested as a decline in network connectedness, in correlation with extreme quantiles. Policymakers can use this information to draft proactive measures, ensuring stability and sustainability in both domestic and international markets.

## 1 Introduction

### 1.1 The world of soybeans

Often referred to as the “king of beans,” soybeans contribute significantly to the global intake of protein, both directly and indirectly [1]. Soybeans stand out as one of the rare plant-based foods offering a complete set of nine essential amino acids [2]. Because of this nutritional profile, soybeans have become a crucial protein source for both humans and animals [3]. The high protein content, ranging from 36 % to 40 %, makes soybeans one of the most protein-dense legumes [4]. Moreover, soybeans are rich in bioactive compounds like isoflavones, which are linked to various health benefits such as reducing the risk of heart disease and cancer [5].

Additionally, their use as a key ingredient in biodiesel manufacturing, particularly in nations such as Brazil, has further heightened their worldwide demand [6]**.** In 2023, soybeans were not only the most traded commodity in the agricultural sector worldwide but also ranked as the fourth most traded commodity overall. None of the top three traded agricultural commodities (corn, wheat, and palm oil) possess the potential to effectively substitute soybeans as a primary source of protein. While corn and wheat do contain protein, their protein content and quality are significantly lower and less efficient compared to soybeans. Furthermore, palm oil does not serve as a protein source, rendering it unsuitable as a substitute for soybeans in terms of protein provision.

This distinction highlights the significant role of soybeans in global trade, reflecting their importance in various industries and economies [7]. After palm oil, soybean oil holds the second position as the most widely used cooking oil worldwide representing 34 % of the value Palm oil market. Its significance extends to global trade, with approximately with a export value of approximately USD 19 billion (10^9^) [8]. The soybean industry is mainly concentrated in a few key countries, primarily the United States, Brazil, and Argentina, which collectively accounted for 90 % of total exported quantity dominating global exports in 2022 [8].

Soybeans have become increasingly vital in meeting the demands of a growing global population. However, the sustainability challenges associated with soybean cultivation necessitate the search for more environmentally friendly food options. The rising production of soybeans contributes to nitrogen pollution due to the extensive use of fertilizers, adversely affecting soil and water ecosystems. The development and adoption of sustainable soybean (RTRS soybean) [9] farming practices could offer a solution to these environmental concerns [3]. The increasing cultivation of soy to meet rising demand has led to the destruction of crucial ecosystems, particularly in the Brazilian Amazon and “Cerrado” regions [1,10]. Currently, around 76 % of soy production is allocated as an affordable, high-quality protein source for animal feed used in meat and dairy production. Approximately 20 % is used in edible oils and food products for human consumption, such as tofu, soy milk, and tempeh. The remaining 4 % is primarily used in industrial applications, mainly for biodiesel production [11].

The global soybean industry, currently valued at USD 155 billion, is expected to reach USD 278 billion by 2031, with an estimated compound annual growth rate (CAGR) of 6 % [12] cited by Ref. [1]. The Food and Agriculture Organization of the United Nations (FAO) forecasted a significant increase in global soybean production for the 2022/2023 harvest season, reaching record levels due to rising demand from China, which is tied to the expansion of livestock production and the replenishment of domestic stockpiles [13].

This significant growth is mainly attributed to the expansion of soybean cultivation areas and improved yields. For instance, soybean production in South America has surged due to a threefold increase in yields and a 200-fold expansion in cultivated land since the 1960s [14]. The soybean sector employs a substantial workforce globally. In 2016, around 240,000 Brazilian farms were engaged in soybean cultivation, while soybean farming in the U.S. alone directly employs around 280,000 farmers [10,15]. The FAO recorded that the soybean production reached 353 million tonnes (Mt) in 2020, a significant increase from about 231 Mt in 2008. This growth was achieved by cultivating soybeans on 126 million hectares of land [13]. Although soybean production consistently grew at a Compound Annual Growth Rate (CAGR) of 3.6 % from 2008 to 2020, this rate declined to 2.42 % from 2014 to 2020 [8]. Despite the decline in the Compound Annual Growth Rate (CAGR) between 2014 and 2020, global soybean production exceeded projections in 2021, reaching 388,098 Mt [16].

Finally, Soybeans are a vital global commodity, offering a complete set of essential amino acids and standing as a primary protein source for both humans and livestock. As the most traded agricultural commodity and a cornerstone of the global economy, soybeans hold immense significance, particularly in the economies of the United States, Brazil, and Argentina, which control 90 % of global exports. Despite the significant nutritional benefits of soybeans, the rapid growth of the soybean industry has raised substantial environmental challenges. These include deforestation in Brazil's Amazon and Cerrado regions, and nitrogen pollution resulting from extensive fertilizer use. Addressing these issues requires robust solutions such as the Round Table on Responsible Soy (RTRS) certification and the exploration of alternative protein sources. With rising global demand pushing the industry's value to a projected USD 278 billion by 2031, it's crucial to balance this growth with sustainable practices to mitigate environmental impacts and safeguard vital ecosystems.

The intricate nexus between the soybean and other agricultural commodities [17], energy commodities [18], and fertilizers stands at the crossroads of contemporary economic research, given their pronounced interdependencies and the escalating concerns about global food security, energy sustainability and commodities financialization [19,20]. As the world grapples with volatile economic conditions, heightened in recent times by geopolitical tensions like the Russo-Ukrainian conflict [21], the global pandemic and Climate Change [22,23], understanding the interrelated dynamics of these critical sectors becomes even more paramount. The recent war has created a new dimension of uncertainty into the global commodities market increasing the connectedness, with agricultural commodities [24]. Given Ukraine's significant role as the “breadbasket of Europe”, experiencing considerable fluctuations in both supply and price determinants. In such a global backdrop, the connectedness and risk spillover mechanisms between these commodities emerge as pivotal constructs. They elucidate how disturbances in one domain, be it due to geopolitical upheavals or other factors, potentially reverberate across others, influencing price determinants, supply chain mechanisms, and global trading systems.

Deploying risk spillover index or connectedness index of [25] based on generalized forecast error variance from a Quantile Vector Autoregressive (QVAR) model, an advanced econometric tool allowing us to track connectedness over time through the examination of extreme quantiles, this study embarks on an exploratory journey to dissect the intricate layers of connectedness or risk of spillover among these pivotal commodity groups in the shadow of geopolitical tensions. Typically, a heightened stress effect elevates the overall connectedness (TCI) within a network. Additionally, the TCI displays significant variations across quantiles [26,27]**.** Recently, numerous research efforts have delved into the relationships between agricultural commodities and factors such as oil shocks, industrial and energy prices, and metal markets using the Connectedness approach [28,29]**.** Many of these investigations utilized methodologies closely aligned with or similar to the approach used in the study. Nonetheless, wavelet techniques and different Copula methods could be considered as potential alternatives.

The risk of spillover index or connectedness index from Ref. [25] based in a QVAR model, with its rich structural underpinnings, allows for a nuanced understanding of the multifaceted interplays at various quantiles, thereby providing insights not just at an aggregate level, although also at specific, and critical junctures of connectedness. By casting a spotlight on these interdependencies, particularly under the specter of the Russian-Ukrainian war, this research aims to provide stakeholders, ranging from policymakers to industry practitioners, with a granular understanding of the systemic intricacies. Furthermore, in discerning the nature and extent of the connectedness among these sectors, the study aspires to inform strategies that mitigate potential vulnerabilities, ensuring a resilient and sustainable future for agriculture, energy, and the broader global economy. Additionally, the interconnectedness and risk propagation among different agricultural commodities fluctuate over time. These variations can be symmetric on some occasions and asymmetric on others, largely due to legislative adjustments in the agricultural sector [30]. Thus, it is vital to comprehend potential asymmetries in price transmission, as well as their underlying causes.

### 1.2 Previous empirical research

The foundation of this investigation is built upon a comprehensive study that previously mapped the international soybean market using a variety of methodologies and analyzed various global historical events. The prior research, conducted by Ref. [31] focused on market cointegration, efficiency, and price transmission across the various international soybean markets. In their methodology, the researchers employed the classic price transmission approach, using the Augmented Dickey-Fuller test (ADF) to determine the order of integration. They utilized ADF with breaks, and the Bai-Perron multiple breakpoint test to pinpoint possible structural breaks within the time series. These breaks were later correlated with exogenous events that had disrupted the market and were thus employed as dummy variables in the cointegration analysis. The authors then performed the Johansen cointegration test, correcting for the previously mentioned dummy variables as a standard procedure. To determine the market's causality, the researchers applied the Granger Causality Test, paving the way for the development of several Vector Error Correction Models (VECM), both in pairs and as a global model. Their findings aligned with those of previous researchers, such as [32,33]. The research of [31] provided insights into market dynamics, price leadership, and overall market efficiency. The authors specifically focused on the events of the US-China trade war, striving to quantify its impact on the international market. The conclusion drawn was unambiguous: the soybean market's development and efficiency exhibit a strong capacity for arbitrage around tariffs, reorganizing trade flows and returning to natural long-term equilibrium. This suggests that the impact of the US-China trade war was insignificant. The investigation also revealed that the Chinese and Argentine markets demonstrated a diminished level of cointegration and, to a certain extent, less market efficiency.

The research by Ref. [34] extended their investigation, building upon previous findings, to search for evidence of asymmetric price transmission within the international soybean market. Their key conclusion was the absence of asymmetric transmission among the markets. However, they identified a methodological challenge related to the different cointegration tests they used. These tests, the Johansen cointegration test, Engle-Granger cointegration test, and the cointegration under asymmetry test by Ref. [35] yielded different results, highlighting methodological limitations. The authors noted that cointegration appeared to be a static concept, highly dependent on selected time windows, and perhaps not entirely suitable to depict the complex dynamics of market situations across varying time periods. Additionally, the price transmission methodology presumed constant parameters, failing to consider potential variations. To address these methodological issues [36], introduced a time-varying approach using the connectedness index based on TVP-VAR. This study corroborated earlier suggestions by Ref. [31] by affirming that a highly mature and developed market can indeed circumvent exogenous shocks. The results displayed a steady connectedness index of 67 % for the last decade. Moreover, no significant evidence was found regarding the impact of the US-China trade war, further validating the conclusions drawn by Ref. [31]. Contrary to prior investigations, the researchers suggested that market causality can be bidirectional, irrespective of price leadership. The authors proposed a shift in leadership, with the historic dominance of Chicago being replaced by shared leadership with Brazil and Argentina [37]. pioneered research on agricultural commodities using the Connectedness Index through the Time Varying Parameter Vector Autoregressive (TVP-VAR) model. They focused on major agricultural commodities such as grains, sugar, livestock, cocoa, corn, soybean oil, lean hogs, soybeans, wheat, and cattle, and energy commodities, specifically crude oil, due to their significant role in global trading. The authors emphasized the key influence of crude oil on agricultural commodities. However, they noted that causality could oscillate from crude oil to agricultural commodities markets, shifting agricultural commodities from the position of net receivers to net transmitters. The study suggested that only grain and livestock demonstrated consistent net transmitter behavior over time. Inspired by the concept of studying connectedness across agricultural commodities. Despite the comprehensive methodologies employed by Refs. [31,34,36,37] which utilize a wide range of Vector Autoregression (VAR) models, cointegration, and Time-Varying Parameter Vector Autoregression (TVP-VAR), these models still fall short in capturing the network of spillovers under extreme scenarios. Exogenous shocks that disturb the market are not reflected at the median of the conditional distribution, although rather, they emerge at the extreme Quantiles of the previously mentioned distribution. To address the aforementioned methodological limitations [38], expanded upon the connectedness methodology. They introduced the use of a Quantile Vector Autoregression (QVAR), which measures connectedness based on quantile levels. This approach involves fitting VAR models at various quantiles, specifically at 0.1, 0.5, and 0.9. and applying it to a broader range of commodities such as soybean, maize, wheat, beef, poultry, coffee grains, cocoa, palm oil, tea, groundnut oil, sugar, rice, and orange juice. The commodities studied included highly interdependent ones like soybean and wheat, the main ingredients in poultry feeds, or split between maize and soybean in the US corn belt, which are primary ingredients in beef production. The authors also incorporated commodities that are unrelated or exhibit no cross-dependency, and hold less transactional significance, such as orange juice and tea. The research uncovered a high connectedness index of over 55 % for the entire study period (1965–2022). This suggests that the interconnectedness among commodities does not solely rely on explicit relationships between them (e.g., Soybean/Feed/Poultry). There may be underlying layers of interconnectedness or complex relationships that are challenging to identify. Interestingly, the authors found that palm oil, corn, wheat, and soybeans are net transmitters, directing the price market of bulk commodities. This finding underscores the importance of soybeans as a price leader in the global agricultural commodity market.

### 1.3 Research gap

Numerous authors have explored the dynamics of the soybean market using various methodologies. Despite the many contributions these authors have made, shedding light on, and deepening our understanding of this topic, a multitude of research questions remain unanswered. This investigation aims to further enhance the general knowledge in this field. Firstly, there is a recognized gap in understanding of the interconnectedness between primary agricultural commodities, energy commodities, and fertilizers.

Agricultural commodities are highly interconnected. This is because the demand for these commodities often treats them as near equivalents, they share comparable costs of production, compete for the same scarce natural resources, and utilize the same market data [39]**.** Additionally, fertilizers, which are critical for agricultural food commodities, represent one of the most significant input costs for arable crops. In a similar vein, crude oil, a crucial energy commodity, is strongly interconnected with agricultural commodities due to its role in production costs. Likewise, natural gas is deeply linked with urea fertilizer due to its significant role in fertilizer production, further demonstrating the intricate web of connections in these sectors. While [37] have already probed this area, there is still room for further research. Their results suggest a bidirectional causality, although their investigation solely included the price of crude oil. The void extends to the pricing of fertilizers, which exhibit cross-dependency with agricultural commodities, such as wheat, maize, barley, and urea, due to their role as crucial inputs in production. Additionally, the relationship between soybeans, soybean meal, and soybean oil has been studied in terms of asymmetric price transmission [40]. However, it remains unclear which commodity holds the market leadership in terms of causality and net transmission. This research aims to address these identified gaps. Even though there might be overlap with the work of [37], this study apply use a Quantile Vector Autoregression (QVAR) methodology, which presents clear advantages over the Time Varying Parameter Vector Autoregressive (TVP-VAR) model. Lastly, the research will seek to understand the influence of the US-China trade war on the soybean market once again, albeit with a broader scope. This includes studying the impact of the Russian-Ukrainian conflict, which has caused shocks in the natural gas and wheat markets. The goal is to trace the origins of these shocks across all commodities studied. The QVAR methodology, adept at identifying hypothetical shocks in agricultural commodities, is highly suited for this purpose. As previously mentioned, implementing quantile regression and fitting VAR models at different quantiles (Q1: 0.1, Q2: 0.5, Q3: 0.9) enables us to identify spillover associated with extreme positive and negative shocks. Furthermore, utilizing the extreme quantiles allows us to ascertain the factors triggering these severe shocks across different agricultural and energy commodities, as well as fertilizers.

## 2 Data & research methodology

The agricultural commodities selection for this study was influenced by an agronomic and an economic cross dependency as well as following the lead of previous studies. Primarily, we focused on arable crops grown on a large scale, choosing those with the highest trading volume and value, such as Soybean, Barley, and Wheat. Additionally, we included byproducts such as Soybean Meal, Soybean Oil, and Sunflower Oil due to their significance in domestic human consumption and animal production, especially in the case of Soybean Meal. The selection of energy commodities, namely Crude Oil and Natural Gas, was driven by their critical roles in agricultural processes. Crude Oil serves as a vital input in arable crop production, whereas Natural Gas plays a key role in the production of fertilizers such as Urea. Finally, we incorporated fertilizers (Urea and DAP) into the study given their crucial impact on crop production. The aim is to understand the relationship between energy commodities and fertilizers, considering the importance of the former in the production of the latter.

The secondary data was sourced from the World Bank and covered a twelve-year period from January 2011 to January 2023 monthly. The data for Soybeans was obtained from the Chicago futures contract (U.S. Soybeans, No. 2 yellow and par, priced in US$ per metric ton) (Soy), acknowledging the Chicago Board of Trade as a credible price reference for the international soybean market. Data for Soybean Meal and Soybean Oil were also derived from their respective Chicago futures contracts (Soybean Meal - Minimum 48 percent protein, and Soybean Oil, exchange approved grades, both priced in US$ per metric ton). For Barley, the study used the spot price of Canadian No.1 Western Barley (US$ per metric ton) as the international price reference. Wheat prices were represented by Kansas Wheat (No.1 Hard Red Winter, ordinary protein, Kansas City, US$ per metric ton), indicating the international prices of wheat. Sunflower oil prices were represented by the spot price of US export from the Gulf of Mexico (US$ per metric ton). Instead of utilizing a specific international price reference for crude oil, the research incorporated a Price Index for Crude Oil (Crude Oil (petroleum), simple average, equally weighed of three spot prices; Dated Brent, West Texas Intermediate, and the Dubai Fateh (Source: World Bank). Similarly, the Natural Gas Index (Laspeyres), average of Europe, US, and Japan (LNG), weights based on 5-year average consumption volumes was employed. The prices of the fertilizers Urea (Ukraine, prill spot f. o.b. Middle East), named USU, beginning March 2022; previously, f. o.b. Black Sea) and DAP (diammonium phosphate), spot, f. o.b. gathered from Bloomberg; Bloomberg L.P., Green Markets (formerly Kennedy Information LLC) named USN.

We adapted the connectedness context according to the methods suggested by Ref. [25]**.** As per [41] approach, the research utilized a quantile vector autoregression to investigate the connectedness between agricultural commodities energy commodities and fertilizer' monthly log return series. These commodities include Soybean, Soybean meal, Soybean oil, Sunflower oil, Barley, Wheat, Crude oil, Natural gas, Urea and DAP. Our analysis focuses on the extreme quantiles lower and upper quantiles. This approach allows us to consider the significant market fluctuations throughout numerous extreme events over the previous 12 years. The formula for the estimation of quantile vector autoregression, QVAR(p) is as follows:

(1) $$y_{t}\left(\tau \right)=\mu \left(\tau \right)+\sum_{j=1}^{p}\phi_{j}\left(\tau \right)\;y_{t-j}+u_{t}\left(\tau \right)$$

where t represents time and τ stands for the quantiles. The vector $y_{t}$ is composed of n endogenous variables, which includes ten agricultural and energetical commodities denoted by u(τ). The matrices of coefficients are represented by $\phi_{j}\left(\tau \right)$ , while the error vector is indicated by $u_{t}\left(\tau \right)$. The upper limit for the lag length, also known as, is 4, a specification that aligns with existing literature by Refs. [41,42].

By leveraging Wold's theorem [43], we adjust the QVAR(p) in equation (1) into a quantile vector moving average form, or QVMA(∞):

$Q_{t}\left(y_{t}|F_{t-1}\right)=\mu \left(\tau \right)+\sum_{i=0}^{\infty }A_{i}\left(\tau \right)u_{t-1}\left(\tau \right)$ with $A_{i}\left(\tau \right)=\Theta_{1}\left(\tau \right)A_{i-1}\left(\tau \right)+\Theta_{2}\left(\tau \right)A_{i-2}\left(\tau \right)+\ldots$ applicable for i=1,2,…; $A_{o}\left(\tau \right)=I_{n}$ and $A_{i}\left(\tau \right)=0$ applicable for $i<0.\;I_{n}$ stands for n×n identity matrix. We applied the QVMA(∞) form and calculate the H-step ahead of generalized forecast error variance decomposition (GFEVD) following equation (2).

(2) $$\psi_{ij,\tau }^{g}\left(H\right)=\frac{\sigma_{jj}^{-1}\sum_{h=0}^{H-1}{\left(e_{i}^{T}A_{h}\left(\tau \right)\Sigma e_{j}\right)}^{2}}{\sum_{h=0}^{H-1}\left(e_{i}^{T}A_{h}\left(\tau \right)\Sigma A_{h}{\left(t\right)}^{T}e_{i}\right)}$$

Σ represents the variance matrix of the vector for error terms, where $\sigma_{jj}$ stands for the standard deviation associated with the error term of j. $e_{i}$ is a vector with n×1 dimensions that assigns the value 1 to the i-th element and 0 to the rest. Following this, the computation of the normalized Generalized Forecast Error Variance Decomposition (GFEVD) [44,45] was carried out. The utilization of GFEVD in this methodology serves as a gauge of robustness, as seen in equation (3).

(3) $$\psi_{ij,\tau }^{∼g}\left(H\right)=\frac{\psi_{ij,\tau }^{g}\left(H\right)}{\sum_{j=1}^{k}\phi_{ij,\tau }^{g}}$$

$\psi_{ij,\tau }^{∼g}\left(H\right)$ illustrates the proportion of forecast error variance in i that is attributed to j when i falls within the τ quantile. Following this, we perform calculations for the subsequent spillover indexes to encapsulate the total spillovers among the variables:

(4) $${FROM}_{i,\tau }\left(H\right)=\frac{\sum_{j=1,j\neq i}^{n}{\psi ͂}_{ij,\tau }^{g}\left(H\right)}{n}\times 100$$

(5) $${TO}_{i,\tau }\left(H\right)=\frac{\sum_{j=1,j\neq i}^{n}{\psi ͂}_{ji,\tau }^{g}\left(H\right)}{n}\times 100$$

(6) $${NET}_{i,\tau }\left(H\right){=TO}_{i,\tau }\left(H\right)-{FROM}_{i,\tau }\left(H\right)$$

(7) $${TCI}_{\tau }\left(H\right)=\frac{\sum_{i,j=1,j\neq i}^{n}{\psi ͂}_{ji,\tau }^{g}\left(H\right)}{n}\times 100$$

The “TO connectedness index,” as denoted in equation (5), indicates the comprehensive influence of variable i on all other variables j. The FROM connectedness index, as outlined in equation (4), conveys the impact of shocks on all other variables j to variable j. The NET connectedness index, referred to in equation (6), measures the net spillovers from i to all other variables j, where a positive (negative) value implies that i acts as a source (recipient) of shocks within the system. Finally, we consider the total connectedness index (TCI). As depicted in equation (7), the total connectedness index (TCI) measures the complete interconnectedness among variables in the system and is employed as a representative indicator for the contagion of market risk.

Our empirical investigation focus on validating the quantile connectedness at the 0.1, 0.5, and 0.9 quantiles. These quantiles represent the interconnectedness among agricultural commodities, energy commodities and bulk fertilizers during extreme negative, median, and extreme positive shifts. Besides studying static interconnectedness, we explore dynamic interconnectedness by calculating the rolling spillover indexes utilizing a 200-day rolling window.

The approach employed in this study presents several distinct benefits over comparable models such as TVP-VAR and DCC-GARCH. Essentially, all these models expand upon the connectedness model [25]. In the QVAR model, the interaction and feedback effects among the variables are contingent on their quantile dynamics. As shocks (either positive or negative) visibly influence extreme quantiles, this methodology is fitting for this type of research. To further validate the results and gain a different perspective, the research also employed a more orthodox alternative methodology. Specifically, the Granger causality test was utilized to map pairwise causality among the various commodities. To achieve this, the following procedure was employed: A VAR (Vector Autoregression) model was created for each pairwise time series. The Lag order selection was studied, and using the Schwarz information criterion, the number of lags was determined for each pair. Subsequently, the Granger Causality Test was conducted, and the results were summarized in a causality matrix.

### 2.1 Methodology limitation

The connectedness approach, particularly when grounded in Quantile Vector Autoregression (QVAR), provides a nuanced framework for understanding market dynamics and the transmission of shocks across different economic sectors. However, it is essential to objectively consider its limitations alongside its strengths to foster a balanced view of its applicability across various market conditions. One notable aspect of the QVAR-based connectedness approach is its pronounced sensitivity to the extremes of market distributions. This characteristic implies a heightened efficacy in mapping out the connectedness and spillover effects under extreme market conditions, such as during bullish or bearish trends, compared to periods of market stability. These give the QVAR approach a substantial advantage to analyse the potential exogenous disruption on the market such as the case of US-China trade war, the outbreak of the pandemic (COVID-19) and the Ukrainian-Russian war. While this feature is invaluable for stress testing and scenario analysis, it suggests a potential limitation in the approach's ability to capture the subtleties of spillover effects during more tranquil market phases.

Acknowledging these limitations does not diminish the value of the QVAR-based connectedness approach in market analysis. Instead, it provides a more comprehensive understanding of when and how the method can be most effectively applied. For regulators, policymakers, and market participants, recognizing these nuances is crucial. It allows for a more informed application of the approach, ensuring that insights derived from QVAR analyses are leveraged appropriately, considering the context of market conditions and the specific objectives of the analysis.

### 2.2 Network chart interpretation

Chart 3, Chart 7, Chart 10 represent the Network Plots for quantile 0.5, 0.1 and 0.9 respectively. The diagrams portray the complex interrelationships between various agricultural commodities across this quantile. Nodes in blue indicate net shock transmitters and nodes in yellow indicate net receivers. The node's size corresponds to the absolute value of the NET connectedness index. The arrows' direction signifies the path of spillover effects between pairs of variables, while the arrows' thickness indicates the strength of these spillovers.

## 3 Results & discussion

### 3.1 Results interpretation

**Chart 1:**
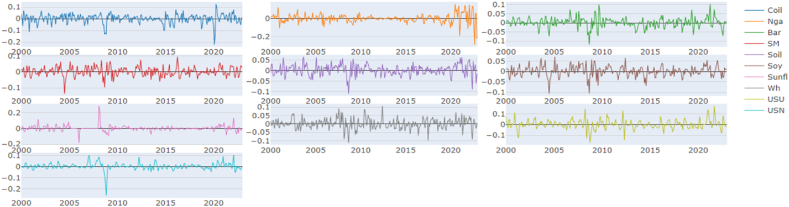
Time series graphical analysis.

The principal findings can be summarized as follows. First, we will present the descriptive statistics (Table 1) and illustrate the time-series data (Chart 1). Secondly, the results will be divided by Quantiles and presented in the following order: the median Quantile (0.5), lower Quantile (0.1), and upper Quantile (0.9). For each Quantile, the corresponding results will be described and interpreted.

**Table 1:** Descriptive statistic of the time series.

	Coil	Nga	Bar	SM	Soil	Soy	Sunfl	Wh	USU	USN
Mean	0.002	0.001	0.001	0.002	0.002	0.002	0.002	0.002	0.002	0.002
Variance	0.002	0.003	0.001	0.001	0.001	0.001	0.001	0.001	0.002	0.001
Skewness	−1.216*	−0.881*	−0.136	−0.296*	−0.313*	−0.467*	2.198*	−0.06	0.178	−2.030*
Kurtosis	4.265*	6.328*	2.343*	1.859*	1.183*	2.073*	20.432*	1.449*	2.743*	20.753*
JB	278.188*	497.991*	64.233*	43.922*	20.661*	59.688*	5041.413*	24.398*	88.277*	5161.001*
ERS	−3.729*	−4.554*	−6.587*	−5.555*	−5.287*	−6.380*	−4.407*	−4.424*	−2.218*	−6.118*
Q(10)	30.222*	33.473*	33.380*	38.852*	34.575*	47.688*	41.278*	15.432*	55.628*	112.851*
Q2(10)	72.629*	115.092*	37.190*	5.873	34.075*	11.143*	35.354*	5.363	28.285*	39.378*

#### 3.1.1 For each quantile

• Total Connectedness Index (TCI): This index measures the total proportion of forecast error variance in a network that can be attributed to shocks from other parts of the network. It provides a sense of the overall interconnectedness within the system.
• Average Dynamic Connectedness (ADC): refers to an average measure of dynamic connectedness over time, an average of the TCI over a specified period, reflecting how connectedness evolves.
• Network Connectedness (NC): reflects the interconnectedness of the entire network or system under study. It quantifies the extent to which components within the network are influenced by each other.
• Net Pairwise Directional Connectedness (NPDC): this measure reflects the net connectedness between two specific commodities in the network. It considers the directional impact of one entity on another and vice versa, offering a net measure of this bidirectional interaction.
• Net Total Directional Connectedness (NTDC): this index measures the net contribution of each individual entity to the total connectedness in the system. It captures the difference between the shocks transmitted by an entity to others and the shocks it receives from the rest of the network.

#### 3.1.2 In overall

• Descriptive statistics:

The Jarque-Bera tests were conducted to assess the normality of the data distribution, while skewness and kurtosis were examined to understand the shape of the distribution. Additionally, extreme quantile ratios were analyzed to identify potential outliers or extreme observations in the dataset.

• Time-series illustration:

A time-series illustration was employed to visualize the trends and fluctuations in the data over a specific period, providing insights into the temporal dynamics of the variables under consideration.

• Chart TCI over various quantiles:

Total Connectedness Index (TCI) is analyzed over various quantiles, it illustrates how the interconnectedness within a financial system or network changes across different states or conditions of that system. This approach is typically used to understand how relationships between different commodities of a network vary under different levels of market stress or volatility.

• Granger causality test:

The Granger causality test was utilized to determine whether past values of one variable can predict the current values of another variable, thus establishing a causal relationship between them.

#### 3.1.3 Variable abbreviations

Each variable is represented by a specific abbreviation to streamline our discussion. “Coil” refers to Crude Oil, a major energy commodity. “Nga” stands for Natural Gas, another key energy source. Agricultural commodities are also included, with “Bar” representing Barley, “SM” for Soybean Meal, and “Soil” denoting Soybean Oil. Additionally, “Sunfl” corresponds to Sunflower, and “Wh” is the abbreviation for Wheat, both important in the agricultural sector. In the realm of fertilizers, we use “USU” to refer to Urea and “USN” for DAP, which stands for Diammonium Phosphate. Finally, “Soy” refers to Soybean price. Each of these variables plays a crucial role in our analysis, representing diverse sectors from energy to agriculture.

### 3.3 Descriptive statistic & graphical illustration

Given that your test result of Jarque-Bera (JB) (Table 1) is larger than any of these critical values (5.99 at alpha 5 %), the null hypothesis at any of these significance levels is rejected. This implies that the sample data does not appear to have the skewness and kurtosis of a normal distribution. Crude oil, Natural Gas, Sunflower Oil, USN (DAP) presented an excess of Kurtosis (Leptokurtic): Distributions with positive excess kurtosis have heavier tails and a more peaked center than the normal distribution. With an excess kurtosis in the data, it indicates the presence of outliers or extreme values. For Barley, Soymeal, Soybean oil, Soybean, Wheat, and Urea they present a negative excess of kurtosis (Platykurtic): Distributions with negative excess kurtosis are flatter with lighter tails compared to the normal distribution. This suggests that the data is platykurtic. Meaning the distribution has fewer and less extreme outliers than the standard normal distribution. The skewness analysis indicates that the time series, in general, exhibits negative skewness, where the tail on the left side (representing smaller values) is longer or fatter than that on the right. However, Urea and Sunflower oil deviate from this trend, exhibiting positive skewness where the tail on the right side (representing larger values) is more pronounced than on the left. Wheat, on the other hand, demonstrated near-zero skewness, suggesting a symmetric distribution, though not necessarily a normal one. The results from the ERS test indicate that the series do not exhibit a unit root, suggesting that it is stationary (Table 1).

### 3.4 Middle Quantile 0.5

#### 3.4.1 Total connectedness index (TCI) (Q 0.5)

The middle quantile, represented as the 0.5 quantile or the 50th percentile, is the median of the distribution. In a quantile regression model, estimating the middle quantile means modeling the median of the dependent variable conditional on the independent variables. The median is less sensitive to outliers compared to the mean. Therefore, the TCI under the median quantile gives a more robust estimate of the central tendency when the data contains outliers or is skewed. In this study based on agricultural commodities, energy commodities, and fertilizers, the TCI under the median (0.5) quantile would help understand the typical (median) connectedness between these commodities under normal market conditions, as opposed to extreme conditions (represented by lower and upper quantiles).

**Chart 2:**
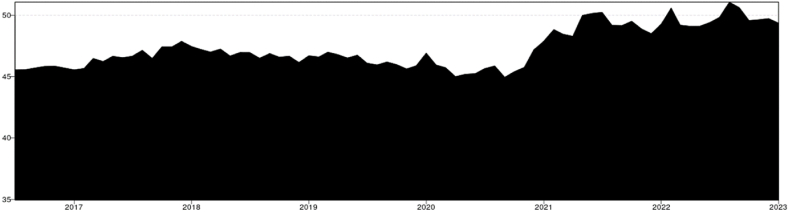
Total connectedness index (TCI) (Q 0.5).

For the 0.5 Quantile the total connectedness averaged around 47 % (Chart 2). From mid-2016 until the end of 2021 it remained stable at 45 %–46 %. Peaking around the beginning of 2021, close to 49 % starting a period of great volatility that remains actively high until the end of the studied period. The influence of US-Chinese trade war seems to be relatively not significant in terms of connectedness around agricultural and energy markets. As previously noted by Ref. [31], this phenomenon may arise due to the market efficiently circumventing the tariff, predominantly impacting the U.S. market (And only soybeans) [46]while benefiting others.

The beginning of the Covid 19 pandemic cannot be correlated with any spike in connectedness or risk of spillover despite that has disturbed in the world trade has not significantly; however, by the end of 2020 the connectedness index started to peak indicating a disruption of the market which might have been a lag effect of the pandemic probably associated with the 200-day rolling window spillover index. The disruptions caused by the pandemic [1] may have impacted the supply chain, leading to positive shocks in prices, particularly evident in the higher quantile Q 0.9. The disruption of the Ukrainian- Russian war (2021 – onwards) it might have a strong influence on increase of shocks spillover, and this is clearly appreciated in the chart showing a greater period of volatility and uncertainty during the before mentioned period. According to Ref. [47] the spillover index peaked during the Russia-Ukraine conflict, with the commodities most significantly impacted being those primarily produced by these nations, including wheat, maize, and barley. The pronounced peak in connectedness within this quantile warrants special attention, particularly since this quantile represents the median. Previous studies utilizing mean models have been inadequate in highlighting external shocks, primarily because such shocks tend to manifest predominantly within extreme quantiles [38].

Timeline & Key events.

1. 2017–2019 (U S.-China Trade War): The trade war between the U.S. and China began in early 2018. Despite significant market disruptions due to tariffs, the impact on the TCI was relatively insignificant. This indicates that global markets adapted efficiently, particularly as the tariffs primarily impacted U.S. soybean markets, with limited spillover effects on other commodities [31,34,36].
2. 2020 (COVID-19 Pandemic):The COVID-19 pandemic began in early 2020. The pandemic did not cause an immediate spike in connectedness. The lack of a significant increase in TCI could be attributed to the swift market adjustments and government interventions that stabilized commodity markets. However, the end of 2020 marked the beginning of an upward trend in connectedness, potentially indicating lag effects of the pandemic on supply chains and market disruptions [15].
3. 2021–2022 (Russia-Ukraine Conflict): The Russia-Ukraine conflict began in 2022. The TCI spiked in early 2021 and maintained higher volatility throughout the period, reflecting significant market disruptions caused by the conflict. Russia and Ukraine are key producers of commodities like wheat, barley, and maize, and the conflict disrupted global supply chains, leading to positive shocks and increased connectedness in these markets. The TCI peaked, highlighting the pronounced spillover effects of the conflict on global commodity markets [1,47].

#### 3.4.3 Average dynamic connectedness (ADC) (0.5 Q)

Table 2 depicts a two-way interaction between different markets. The system's interconnectedness is broken down into two categories. Firstly, the final column, labeled “FROM,” represents the total aggregate of shocks received by all the markets under consideration from the entire system. The calculation for this column's value is the sum of all the horizontal figures, excluding the diagonal figures. Secondly, the “TO” row at the end illustrates the shock distribution from each time series to the entire system. The total for this row is determined by adding all the vertical figures, excluding the diagonal ones. The numbers represented column-wise signify the contribution of one commodity to the forecast error variance in another one. The numbers provided row-wise depict the cumulative contribution of all other commodities to the forecast error variance in a specific commodity.

**Table 2:** Average dynamic connectedness (ADC) (0.5 Q).

	Coil	Nga	Bar	SM	Soil	Soy	Sunfl	Wh	USU	USN	FROM
Coil	66.4	2.01	6.95	2.38	8.92	5.13	2.29	0.96	1.48	3.47	33.6
Nga	3.91	83.76	2.21	2.21	1.98	1.9	1.31	0.67	0.82	1.22	16.24
Bar	4.81	0.69	41.04	12.66	9.94	16.37	2.2	11.4	0.63	0.25	58.96
SM	1.08	0.94	9.7	36.91	10.04	30.53	1.42	8.76	0.32	0.3	63.09
Soil	4.87	0.4	9.53	8.39	43.63	21.36	2.66	7.25	0.93	0.99	56.37
Soy	1.66	0.41	10.55	24.48	19.83	32.19	1.66	8.42	0.59	0.21	67.81
Sunfl	2.58	0.42	3.52	2.27	5.48	3.58	77.64	0.99	0.45	3.07	22.36
Wh	0.83	1.17	11.14	12.41	8.41	14.15	0.9	50.49	0.14	0.35	49.51
USU	3.27	4.1	2.1	1.37	2.38	1.92	2.93	2.02	73.67	6.23	26.33
USN	5.19	2.89	2.51	2.31	3.6	2.76	3.31	1.68	7.19	68.55	31.45
TO	28.21	13.02	58.2	68.49	70.58	97.71	18.7	42.16	12.55	16.1	425.71
Inc.Own	94.61	96.79	99.24	105.39	114.21	129.9	96.34	92.65	86.22	84.65	TCI = 49 %
NET	−5.39	−3.21	−0.76	5.39	14.21	29.9	−3.66	−7.35	−13.78	−15.35	
NPT	4	3	5	7	8	9	3	5	1	0	

The first noteworthy observation when we rank commodities by the magnitude of their spillover transmission to other markets, is that Soybean stands out as having the most significant influence in terms of shock spillover within the entire market studied. With 97.71 % of spillover transmission, Soybean emerges as the top net transmitter (29 %) and price leader. This outcome aligns with the research conducted by Ref. [38] which underscored the paramount role of soybean as a net transmitter. However, it contrasts with the findings of [37] which highlighted crude oil's key influence over soybean. This dominant position of soybeans in the international agricultural commodities market can be correlated to their vital role in global trade. As the most traded agricultural commodity and the fourth most traded commodity worldwide [7], resulting in soybeans take position as net spillover transmitter in the market.

The second observation when ranking commodities by the extent of spillover transmission reveals that, following Soybean in first place, its byproducts Soybean Oil and Soybean Meal take the second and third positions, respectively. This can be seen as a further testament to the primary role and dominance of Soybean. Soybean Oil has been observed to transmit approximately 70.58 % of spillover, with a net transmission of 14.21 %. Subsequent to this, Soybean Meal demonstrates an overall transmission of 68.49 %, and a net transmission of 5.39 %. The triumvirate of Soybean, Soybean Oil, and Soybean Meal are the only three commodities with a positive transmission balance, in other words, they are net transmitters. This suggests that for the 0.5 Quantile, these commodities have dominated the market, acting as price leaders, and playing a key role in price formation under “normal” market circumstance (Q 0.5). The significant role of soybean oil and soymeal in the market can be attributed to their interdependent economic relationship, as both are by-products of soybeans. This interdependence means that the demand for soybean oil and meal directly influences the demand and price of soybeans, which in turn are the primary cost factors for soybean oil and meal production. The joint products theory suggests that the price of soybeans is determined by a combined average of the earnings from soybean meal and soybean oil, subtracting the costs involved in processing [48]. revealed that these theories do not necessary apply for this case. According to the researchers the constant in the cointegration equation, which was expected to align with theoretical predictions, did not. This constant shows that the price of soybeans in Rotterdam is higher than the combined average prices of Dutch oil and Hamburg meal. This suggests that the price of Rotterdam soybeans does not have a direct correlation with the prices of the Hamburg meal and Dutch oil, indicating a lack of joint product relationship between them. Furthermore [48]. findings from the Vector Error Correction Model (VECM) suggest that over the long term, only the price of soybeans adjusts to maintain equilibrium. This makes sense considering that oil and meal are products with significantly larger markets. Soybeans are among the most influential commodities, notably for the 0.5 Quantile (Normal market conditions). Intuitively, this commodity has the most significant impact on soybean meal, at 30.53 %, and soybean oil, at 21.36 %. However, the reverse influence (that is, from soybean meal and soybean oil to soybeans) is 24 % and 19.83 %, respectively. This indicates a strong bidirectional causality, with soybeans predominantly leading in price fluctuations. In contrast with [48] findings our results suggest that the price of soybeans is a primary component influencing the price changes of both soybean oil and meal. This shifts the conversation's focus, suggesting that price formation is primarily driven by production costs. However, the strong bidirectional causality also implies that price formation could be influenced equally by both cost and demand factors.

Our research suggests there is a potential for multidirectional spillover effects among these commodities, arising from either the cost side or the demand side. Furthermore, this close relationship could accelerate the system's adjustment speed, mirroring the price fluctuations of the leading commodity (soybeans) rapidly. This rapid adjustment may lead to a perceived, yet misleading, causality in price movements (or price spillover) from secondary commodities (such as soybean meal and oil) to the broader market.

The second most noticeable spectrum of influence for soybean is the influence of this commodity on Barley (16.37 %) and Wheat (14.15 %) with the inverse influence being considerably lower for Barley at 10.55 % and Wheat at 8.4 although still bidirectional. The third spectrum of influence of soybean is considerably lower Crude Oil (5.13 % v 1.66 %), Sunflower oil (3.58 % v 1.66 %), Phosphate fertilizer (2.76 % v 0.21 %), Urea (1.92 % v 0.59 %), Natural Gas (1.9 % v 0.41 %) this means that the connectedness with this last group is considerably lower that from the beforementioned first and second, despite soybean still influence the price. Finally, soybean is mostly influenced by itself (32.19 %).

Barley, ranking fourth (58 %) and Wheat, in fifth place (42 %), are respectively noted. However, with their negative net balances of −0.76 % and −7.35 % respectively being identified as net receivers, it becomes clear that Barley and Wheat are predominantly price followers in the agricultural commodities market. The relatively secondary position of Wheat and Barley in the agricultural commodity market could be attributed to their significantly lower total trading volumes [7] compared to soybeans.

Following Crude Oil (28.21 %), Sunflower Oil (18.72 %), Natural Gas (13.02 %), Urea (12.55 %), and Di-ammonium Phosphate (DAP) (16.1 %) demonstrate minor roles in price leadership. These commodities, classified as net receivers and price followers, are influenced to a larger extent than they influence others. For the case of Crude Oil the results are in line with [49] that using the connectedness approach show that the influence exerted by the crude oil market on the returns of agricultural commodities was less pronounced than the influence in the reverse direction, where agricultural commodity returns impacted the crude oil market. For the case of Sunflower Oil [50,50] indicated a strong interconnectedness between various vegetable oils, particularly noting that sunflower oil prices are highly sensitive to changes. This implies that factors affecting the sunflower oil market are not solely internal but are also affected by fluctuations in other vegetable oil markets. These might explain the net receiver's behavior of the sunflower market as well with the Russia-Ukraine conflict has had far-reaching effects on global trade dynamics. Specifically, it has resulted in a notable decrease in sunflower oil exports. This decline can be attributed to the closure of plants and ports in Ukraine, a major sunflower oil producer [51]. Consequently, there has been a surge in demand for alternative oils, particularly soybean oil, as market participants seek to fill the gap left by the disruption in sunflower oil supply. This shift in demand has not only altered trade flows but has also underscored the interconnectedness of global commodity markets.

These results are highly intriguing, as they reposition energy commodities and fertilizers (Natural Gas, Crude Oil, and Fertilizer) to a secondary level, driven predominantly by certain agricultural products. The Natural Gas and Crude Oil net spillover receivers’ behavior at normal market conditions (Q 0.5) is difficult to explain. Firstly, despite the trading contract volumes for natural gas surpassing those of soybeans and all other commodities examined in this study [7], this does not necessarily confer a leading position in the market or establish it as a net transmitter of price behavior. However, these results align with the findings related to crude oil spillover, as previously mentioned by Ref. [49], who assessed the behavior of crude oil market spillovers and their impact on agricultural commodity markets.

#### 3.4.4 Network connectedness middle quantile (NC) (Q 0.5)

**Chart 3:**
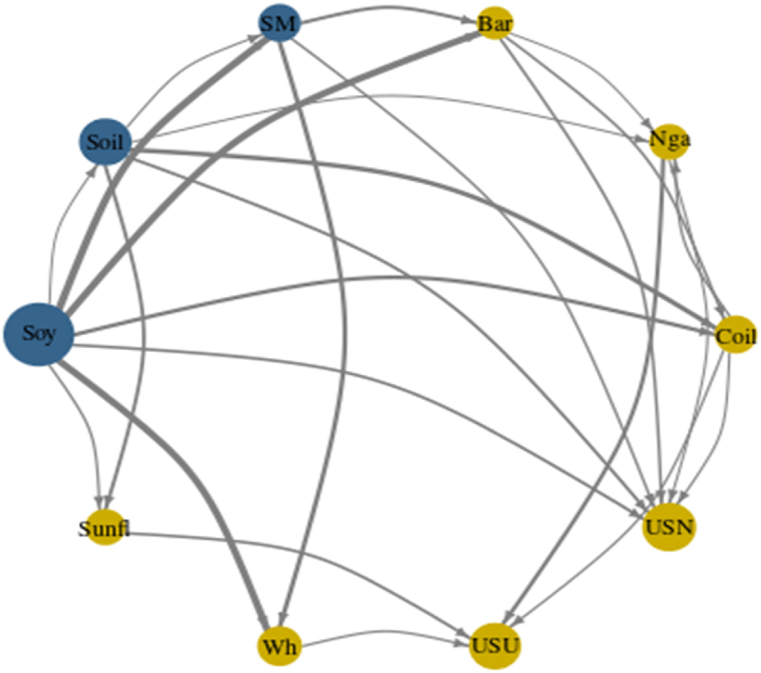
represents the Network Plot for quantile 0.5. As before mentioned, soybean plays the leading the studied market, being the main net transmitter followed by Soybean oil and Soybean meal. Together the three the only net transmitting leading the shocks spillover in the market Phosphate fertilizer and Urea the most susceptible commodities and net receivers.

Chart 3 Network connectedness (NC) (Q 0.5).

#### 3.4.6 Net pairwise directional connectedness middle quantile (NPDC) (Q 0.5)

**Chart 4:**
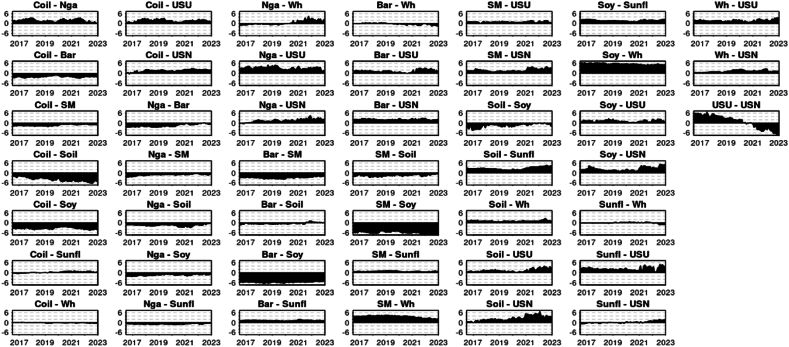
Net pairwise directional connectedness (NPDC) (Q 0.5).

Chart 4 illustrates the Net Pairwise Directional Connectedness for the median Quantile, 0.5. This graphical representation allows us to interpret the direction of the spillover between pairs. A clear observation from the chart is the dynamic within the pair of crude oil and natural gas. Here, crude oil consistently shows a causal influence, transmitting shocks to natural gas throughout the entire period under consideration. This relationship was previously studied by Ref. [52] finding that the Crude oil prices and taxation have significant impacts on natural gas prices, suggesting a unidirectional relationship from crude oil to natural gas prices (Under normal market circumstances Q 0.5) In line [53] research unraveled a unidirectional causality runs from the Dubai crude oil market to the US natural gas market, with the Dubai crude oil price positively affecting the US natural gas price.

A similar pattern can be seen between crude oil, urea, and phosphate, indicating that crude oil's price significantly influences the price of these fertilizer and energy commodities (Under typical market conditions, (UTMC) Q 0.5). This is in line with [54] research that investigated the time-varying price spillovers between natural gas and crude oil markets. While it focuses on natural gas and crude oil, the findings highlight the interconnectedness of energy markets, which could indirectly suggest similar dynamics might exist between crude oil and urea prices, given the energy-intensive nature of urea production (UTMC-Q 0.5).

The net pairwise directional connectedness amongst fertilizers, specifically urea and phosphate, presents an intriguing outcome. Until mid-2021, urea was the primary transmitter, while phosphate primarily received shocks. However, this dynamic experienced a reversal later on, with phosphate assuming the role of the main transmitter and urea becoming the primary receiver of shocks. The economic dynamics between urea and phosphate fertilizers are influenced by their production processes and global supply chains. Urea, a nitrogen fertilizer, is predominantly produced through the Haber-Bosch process, which heavily relies on natural gas, making its production dependent on natural gas price. Phosphate fertilizers, on the other hand, are derived from mined minerals, with major production concentrated in countries like China, the United States, India, Morocco, and Russia. This geographical concentration in production and the different base resources required for urea and phosphate fertilizers create a complex global trade dynamic, where disruptions in supply or geopolitical tensions can significantly impact global prices and availability. The Ukrainian-Russian conflict may limit Russia's ability to export phosphate, leading to reduced supply and increased prices for this commodity. Consequently, this could cause price rises in related commodities, such as urea, due to spillover effects. Prior to Russia's military action in Ukraine in February 2022, the world was already experiencing a surge in fertilizer costs. This increase was a consequence of the COVID-19 pandemic, which led to widespread disruptions in supply chains and transportation issues, affecting global fertilizer production and distribution [55]. By August 2021, prices for most fertilizers had risen by 25 percent compared to March of the same year. The situation was further exacerbated by Russia's incursion into Ukraine early in 2022, which caused more disruptions in transportation, particularly in the Black Sea area, and led to the imposition of additional trade barriers. These developments further constrained the already limited supplies of fertilizer, resulting in a price spike of over 50 percent between February and April 2022 [55].

A study by Ref. [56] specifically investigates the causal relationships among the prices of various fertilizers, including urea, diammonium phosphate (DAP), muriate of potash, rock phosphate, and triple super phosphate. The results show that the urea price Granger causes all other fertilizer prices, including DAP. This indicates a significant causal relationship where changes in urea prices can predict changes in DAP prices. Our findings are consistent with [56] research up to 2021, after which we detected a shift in causality using the connectedness approach, potentially cause by the before explained geopolitical situation [57]. study indicated that the recent global financial crisis had a significant impact on the cointegration and causal connections between fertilizer markets. Moreover, the crisis also influenced the asymmetry, leverage effects, and the enduring nature of shocks on fertilizer price volatility. The Ukrainian-Russian war might affect the casual connection between DAP and Urea inversing the relationship. Sanctions and trade constraints have reduced the export of fertilizers from Russia and Belarus, major players in the global production of nitrogen and potassium fertilizers. This has caused scarcities and a sharp rise in fertilizer costs. As a result, numerous farmers have transitioned from wheat to crops that require less fertilizer, such as soybeans, adding to the unpredictability in global food markets [1].

Another notable shift in causality was observed between Natural Gas and Wheat. Until 2020, Wheat was the dominant net transmitter, although Natural Gas assumed this role thereafter. This change may have been influenced by the occurrence of the Russian-Ukrainian war. This can be explained given that fertilizers are a significant input in wheat production, this relationship suggests that rising natural gas prices can indirectly impact wheat prices through increased production costs.

Similarity the Barley shift in Wheat's net directional causality could potentially be a consequence of Russian attacks on Ukrainian grain facilities, which hindered Ukraine's ability to export their wheat production. The Barley causality in Wheat was already reported [58] indicating that developments in the barley market can significantly influence wheat market prices. The Barley shift in importance have been previously reported by Ref. [47]. As a result, the shift in causality led to the transmission of shocks from wheat prices to barley prices.

Soybean is unanimously the leading commodity across all studied pairs, always serving as the net transmitter for other commodities. The degree of directional connectedness is more pronounced among grains than energy commodities or fertilizers. This might imply that spillover primarily initiates from soybean to the grain market before reaching others such as energy commodities and fertilizers. Alternatively, it could indicate that shocks are more intensely transmitted between commodities with high cross-relatedness, such as grains. The data clearly underscores the significance of soybean in terms of causality and market leadership.

#### 3.4.7 Net total directional connectedness (NTDC) middle quantile (Q 0.5)

**Chart 5:**
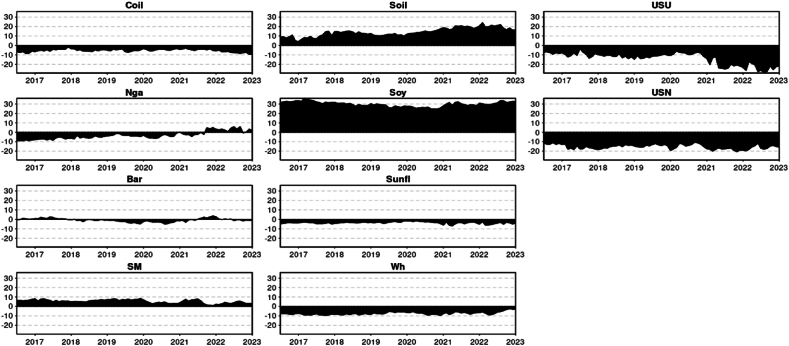
Net total directional connectedness (NTDC) (Q 0.5).

The Net Total Directional Connectedness provides an indication of the spillover direction for a specific commodity over the study period. If the sign is positive, this signifies that the commodity is a net transmitter of influence, whereas a negative sign indicates that the commodity is a net receiver. For the 0.5 Quantile (representing normal market condition) in line with [49] and in contrast with [37] for the studied market, crude oil has consistently been a net receiver throughout the entire period (Chart 5). Conversely, and in line with [38] soybeans have persistently acted as a net transmitter. Soybean oil and soybean meal have displayed a similar pattern, albeit to a lesser extent. Natural gas was a net receiver up until the end of 2021, which may be correlated with the dispute between Russia and the European Union. Wheat has mostly behaved as a net receiver throughout the entire period, though a potential shift began in mid-2022. However, it is still too early to confirm this trend. Both urea and phosphate fertilizer have consistently acted as net receivers during the studied period. However, beginning in the first quarter of 2021, urea exhibited a heightened vulnerability to external shocks, likely stemming from the impact of the Ukrainian-Russian gas dispute on natural gas prices. Lastly, barley has fluctuated throughout the studied period, alternating between being a net receiver and transmitter of influence.

### 3.6 Lower Quantile (Q 0.1)

#### 3.6.1 Total connectedness index (TCI) (Q 0.1)

In quantile regression, analyzing the TCI at the lower quantile, specifically the 0.1 quantile or the 10th percentile, offers unique insights, particularly in understanding the behavior of a dependent variable under less typical conditions. This quantile focuses on the lower end of the distribution of the dependent variable. The 0.1 quantile TCI enables understanding how variables behave in terms of connectedness under less favorable or more extreme lower-end conditions, such as market downturns, low demand, or low prices. In the context of our research on agricultural and energy commodities, the TCI under 0.1 quantile allows you to understand the dynamics of these commodities during periods of significant market stress or downturns.

**Chart 6:**
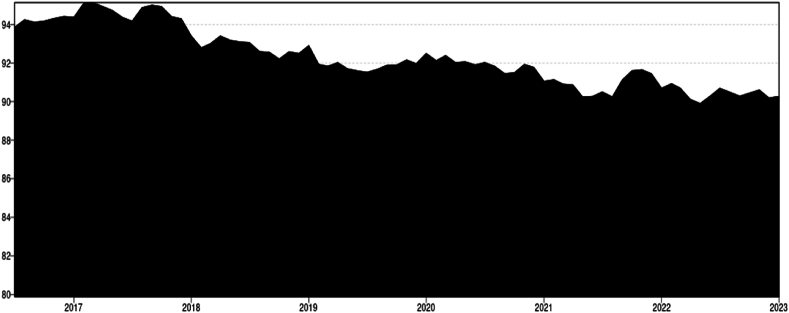
Total Connectedness index (TCI) (Q 0.1).

The total connectedness for the lower Quantile stands at 91 %, indicating a substantial level of interconnectedness across the different commodities, the highest among the varying Quantiles. The overall trend exhibits a consistent decrease in connectedness over the studied period. Despite significant global events such as the US-China Trade War in January 2018, the outbreak of a global pandemic in 2019, or the Ukrainian-Russian conflict in February 2022, the lower quantile connectedness seems to have decreased and stabilized over time. (Chart 6). The diminished connectedness observed in the lower quantile correlates with negative price shocks. During events such as the pandemic and the Russo-Ukrainian conflict, it is plausible that the predominant external disturbances were positive shocks. These shocks primarily impacted the upper quantile, inducing inflationary pressures. These results align with those of [38], who employed a similar methodology over a more extended period. Their study revealed that the connectedness within the agricultural commodities market consistently increased in the first quantile (Q1) from 1976, peaking during the subprime crisis of 2008-9. However, since 2018, there has been a steady decline in the system's connectedness.

Timeline & Key events.

1. 2017–2018: The TCI peaks in early 2017 and maintains a relatively high level throughout 2017 into early 2018. This period saw the beginnings of trade tensions between the U.S. and China, impacting commodity markets, including soybeans and other agricultural products. The peaks in TCI suggest heightened interconnectedness among commodities during this period, possibly due to market anticipation and reaction to trade policies.
2. 2018–2019: A dip in TCI occurs around mid-2018, coinciding with the escalation of the U.S.-China trade war. The trade conflict led to significant tariffs on various commodities, including soybeans, which were especially impacted due to China's role as a major buyer. This dip might reflect reduced interconnectedness as the soybean market and other commodities responded uniquely to trade measures.
3. 2020 (COVID-19 Pandemic): The TCI fluctuates and generally trends downward through 2020, which aligns with the onset of the COVID-19 pandemic. The global disruption caused by the pandemic affected supply chains, demand, and production across commodities, potentially leading to more isolated movements rather than synchronized trends.
4. 2021–2022 (Post-Pandemic Recovery and Russia-Ukraine Conflict**)**: In 2022, following a period of fluctuation throughout 2021, the Total Connectedness Index (TCI) experienced a notable decline. This dip can be attributed in part to the repercussions of the Russia-Ukraine conflict on global commodity markets. The conflict led to significant disruptions in key sectors such as wheat, oil, and energy, likely diminishing overall interconnectedness, particularly among the lower quantiles. Conversely, there was an observed increase in connectedness among the higher quantiles, notably within the 0.9 Q (Positive Shocks) range. Additionally, the lingering inflationary effects of the pandemic contributed to price hikes, particularly evident in the upper quantile.

Overall, the TCI (Q 0.1) chart shows fluctuations that appear to align with major global economic events, reflecting changes in the interconnectedness of commodity markets, especially during periods of extreme market conditions like trade wars, pandemics, and geopolitical conflicts.

#### 3.6.3 Average dynamic connectedness (ADC) lower quantile (Q 0.1)

Table 3 presents a bi-directional interplay among various markets. The commodity that proves to be the most influential for the 0.1 Quantile is Soybean, displaying a spillover transmission of 105 % and a net average dynamic connectedness (ADC) of 21 %. It is closely followed by Soybean oil, which exhibits a spillover transmission of 99.72 % and a net ADC of 16.91 %. This results in contrast with [38] for the Q1 that showed on the first place and second place as in terms of net spillover on the system was held by Palm oil and wheat respectively. This discrepancy can be attributed to the significantly longer research period in Ref. [38] study compared to ours. Considering that the prominence of soybean has only emerged in the last 20 years, and [38] research spanned the past 63 years, the importance of soybean becomes apparent only in the most recent two decades. The increasing importance of soybeans over the last 20 years can be attributed to several factors, including its role as a substitute product, the socio-economic impacts of its trade, and the influence of agricultural policies and market demands [59].

**Table 3:** Average dynamic connectedness (ADC) (0.1 Q).

	Coil	Nga	Bar	SM	Soil	Soy	Sunfl	Wh	USU	USN	FROM
Coil	17.56	7.95	10.18	9.1	11.44	10.24	5.73	9.4	8.84	9.57	82.44
Nga	9.5	21.92	8.88	9.4	9.38	10	6.57	8.8	7.84	7.71	78.08
Bar	9.63	7.91	14.08	10.34	11.38	11.88	6.23	11.23	9.18	8.13	85.92
SM	7.44	6.9	11.29	18.23	10.96	16.64	5.6	11.25	6.26	5.42	81.77
Soil	9.43	7.07	10.72	10.44	17.19	13.8	6.17	10.3	7.65	7.22	82.81
Soy	8.04	6.59	11.58	13.94	13.32	16.21	5.57	11.23	7.5	6.02	83.79
Sunfl	9.86	8.22	9.41	8.83	10.8	9.89	16.07	9.23	8.71	8.98	83.93
Wh	8.18	7.37	11.56	11.97	10.7	12.31	5	19.31	7.05	6.55	80.69
USU	11.77	8.58	9.26	8.39	11.02	9.6	6.23	9.7	16.01	9.43	83.99
USN	10.72	9.05	10.11	9.64	10.72	10.62	6.37	9.77	9.96	13.05	86.95
TO	84.55	69.66	93	92.06	99.72	104.97	53.47	90.91	73	69.02	830.36
Inc. Own	102.11	91.58	107.08	110.3	116.91	121.18	69.54	110.22	89	82.07	TCI = 91 %
NET	2.11	−8.42	7.08	10.3	16.91	21.18	−30.46	10.22	−11	−17.93	
NPT	4	3	7	6	8	9	0	5	2	1	

While the specific influence of soybeans on the 0.1 quantile of agricultural commodities is not directly addressed in literature review, previously collectively suggest that soybeans, as a key agricultural commodity, have a significant impact on market dynamics, particularly in extreme market conditions [17].

For this Quantile, Barley, with an ADC of 93 %, supersedes Soymeal, which has an ADC of 92 % and a net ADC of 7.08 %. Barley transitions from being a net receiver (Q 0.5) to a being net transmitter (Q 0.1). This finding is in line with [47] suggested that the peak of spillover effects during the Russia-Ukraine conflict significantly impacted commodities produced in those countries, as evidenced by the cases of barley and wheat.

Despite being displaced by Barley, Soybean Meal maintains its position at fourth place overall, although it ranks third in terms of net ADC 10.22 %. Both Wheat (ADC 90, Net 10.22) and Crude Oil (ADC 84.6 %, Net 2.11) shift from their previous roles as net receivers to net transmitters, providing a stark contrast to the trends observed in the middle Quantile. These results uncover the potential of Barley, Wheat and Crude Oil to shift from net receivers to net transmitter within the lower and medium quantile. In simpler terms, our findings highlight the capacity of these three commodities to propagate spillover effects during economic downturns.

In the lower Quantile, Soybean again claims the top spot as the most influential commodity. However, the magnitude of influence in terms of risk of spillover for this Quantile has reduced for Soybean Oil (13.8 %) and Soybean Meal (16.64 %), in comparison to the medium Quantile (21.36 % and 30.53 %), this finding is in line with [38]. This pattern is also mirrored in the second sphere of influence, which includes Barley (11.8 %) and Wheat (12.31 %). In contrast, the third spectrum, comprising Crude Oil, Natural Gas, and Fertilizer, exhibits an inverse pattern. Here, the influence of Soybean significantly escalates, leading to a substantial increase in the magnitude of the negative shocks transmitted to Crude Oil, Natural Gas, and Fertilizer. As previously mentioned, Crude oil has significantly increased its total spillover transmission to other commodities, resulting in the highest absolute spillover to fertilizers like Urea (increasing from 3.27 % to 11.77 %) and Phosphate (rising from 5.29 % to 10.72 %). The influence of Crude oil on different commodities has risen within the 0.1 Quantile. Crude oil has notably shifted from being a net receiver to a net transmitter of negative shocks. Demonstrating a similar pattern, Wheat and Barley have also experienced a shift from being net receivers to becoming net transmitters. Overall, within this Quantile, the interconnectedness is considerably higher. As a result, the transmission of shocks has also increased across all different commodities within the studied sample. However, only some have managed to turn the net difference into a positive balance, transitioning from being net receivers to net transmitters (Table 3).

#### 3.6.5 Network connectedness (NC) lower quantile (Q 0.1)

**Chart 7:**
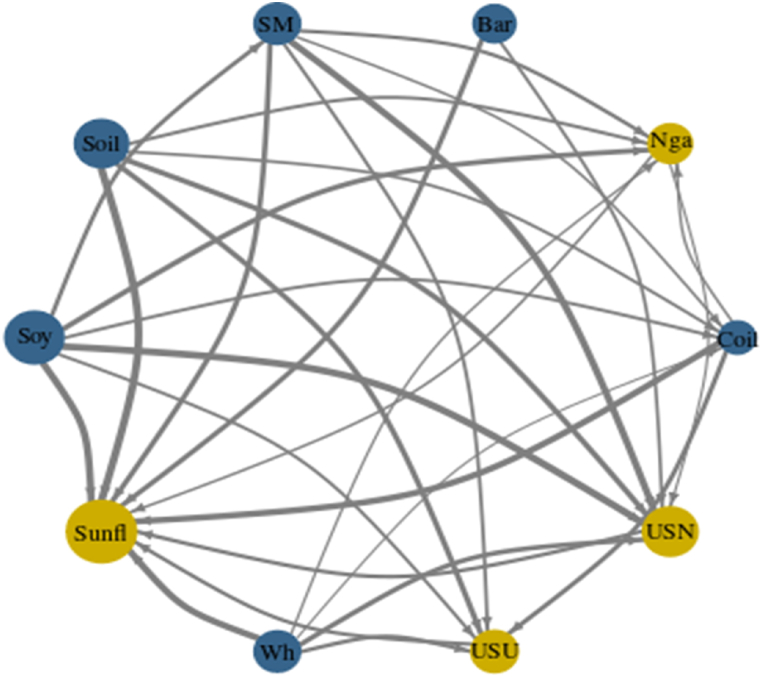
Network connectedness (NC) (Q 0.1).

Chart 7 illustrates the Network Plot for the 0.1 quantile, representing the intricate interconnections among different agricultural commodities within this range. For this quantile, which represents negative shocks; Soybeans and Soybean sub-products continue to dominate as leading net transmitters. This trend is not evident in Ref. [38] investigation, where soybeans, wheat, and palm oil equally dominate. This discrepancy could be due to the longer time span covered in their research.

It becomes evident that Crude Oil has also started to assume a transmitter role following the line of [37,37]. Barley and Wheat have emerged as the second most influential group following Soybeans and Soybean sub-products [47]. has pointed the same regarding the growing influence of Barley post Pandemic and during the Ukrainian and Russian war. Additionally, Sunflower oil has seen a drastic surge in its position as a net receiver, receiving substantial influence from almost all commodities in line with [50].

#### 3.6.7 Net pairwise directional connectedness (NPDC) lower quantile (Q 0.1)

**Chart 8:**
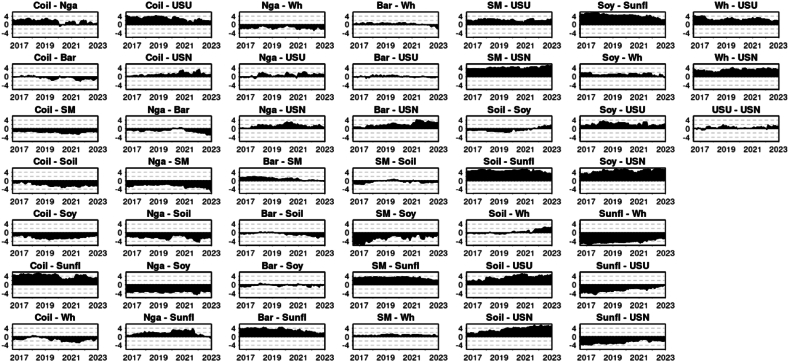
Net pairwise directional connectedness (NPDC) (Q 0.1).

From the Net Pairwise Directional Connectedness chart (Chart 8), it can be inferred that soybean generally holds the most influence position within the lower Quantile of commodities. This finding contrast with [60] that investigated the return and volatility spillover among agricultural commodities and emerging stock markets, including soybean. They identified soybean as a large recipient of spillover over time, indicating it may not predominantly act as a net transmitter in this scenario. The variance in results could be attributed to the distinct methodology employed by Ref. [60], specifically the Global Vector Autoregressive (GVAR) model. Additionally, the inclusion of stock prices from various futures markets in their sample might have contributed to the differences observed.

However, this influence can vary depending on the rolling windows being considered. For instance, the Barley-Soybean pairwise directional connectedness fluctuated notably at the start of 2018, and again in 2021, with soybean regaining its dominant role afterwards. This transient emergence of barley as a significant transmitter of shocks was previously detailed by Ref. [47]. The same is clearly observed in the pair Crude Oil and Barley where a shift on causality happen around mid-2018, emerging Barley as the main shocks transmitter. Interestingly [61] found a strong transmission effect from oil only in the tail quantiles (Q1 and Q2) in longer time horizons, particularly for barley. This suggests that barley prices may be affected by oil in periods of increased market turbulence. In summary, the relationship between soybeans and barley seems to remain stable with occasional fluctuations likely due to changes in trade, weather, global demand, and energy prices.

A similar pattern is observable in the Soybean Oil-Soybean pairing. Here, soybean has typically been the primary shock transmitter until 2022, when soybean oil began to exert greater influence over soybean, this last behavior is in line with [48] findings. Furthermore, soybeans played a leading role in transmitting shocks to wheat. However, as of the start of 2023, wheat has begun to surpass soybean in terms of net shock transmission. This last fact might be related with the influence of Russian-Ukranian war as before explained by Ref. [47]. In summary, the stable relationship between soybeans and soybean oil reflects the direct processing link, but fluctuations arise due to factors like biodiesel demand, trade policies, and global vegetable oil market shifts.

Upon examining the energy commodities more closely, for the considered Quantile, Crude Oil predominantly led the spillover transmission over Natural Gas for almost the entire research period, barring an early peak when the net transmission was briefly positive for Natural Gas over Crude Oil. Crude Oil maintained its position as the chief conduit for spillover transmission to both energy commodities and fertilizers. This phenomenon can be considered time varying depending time windows selected for example [54] for the period 1994 to 2014 studied time-varying price spillovers between natural gas and crude oil markets, finding that, contrary to earlier research, natural gas prices led the price of crude oil with spillover effects lasting up to two weeks. However, after 2006, the price dependencies between these two energy commodities weakened, suggesting increased independence in price determination. In summary, the relationship between crude oil and natural gas generally remains stable due to their interconnected roles in the energy sector. Fluctuations arise from substitution effects, production dynamics, and global economic or geopolitical factors.

Natural Gas, meanwhile, proved to be a consistent net transmitter of shocks to fertilizers, specifically Urea and Phosphate, throughout the entire period. While direct research on the spillover effects of natural gas prices on urea prices is limited, the existing studies emphasize the critical role of natural gas as a primary input in urea production. This suggests that fluctuations in natural gas prices are likely to have a significant impact on the production costs and market price of urea [62]. In summary, natural gas has a direct and strong influence on urea due to its role in production, while it influences phosphate fertilizers more indirectly through overall energy costs and market factors.

Although there is a low degree of directional connectedness between the fertilizers Urea and Phosphate within the lower Quantile, Urea emerges as the net shock transmitter in line with [56].

#### 3.6.9 Net total directional connectedness (NTC) lower quantile

**Chart 9:**
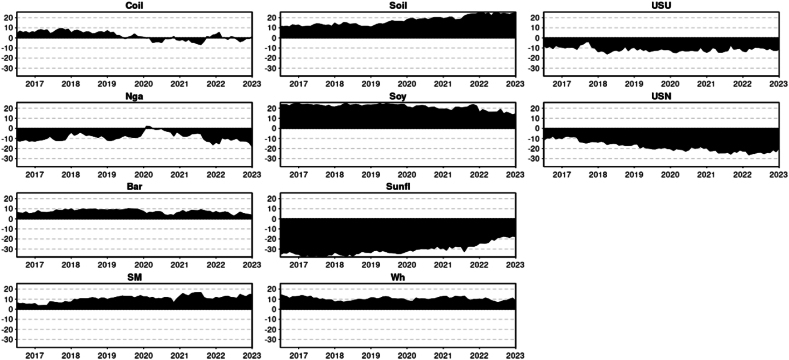
Net total directional connectedness (NTDC) lower Quantile (Q 0.1).

The Total Directional Connectedness for the lower Quantile (Chart 9) demonstrates a shift in the behavior of crude oil around mid-2019 This finding are in line with [37] Crude oil not only influences other commodity markets, but it is also equally affected by other commodities innovations. Crude oil began to alternate roles between acting as a net transmitter and a net receiver of shocks. Prior to this period, crude oil was consistently classified as a net transmitter. This represents a stark contrast to its behavior within the middle Quantile, where it was predominantly characterized as a net receiver. This can be clarified by stating that during market turbulence, crude oil can either transmit volatility to other commodities or absorb it from them. For this lower Quantile, Natural Gas, Urea, Phosphate, and Sunflower oil function as net recipients of spillovers. Contrary to their behavior in the middle Quantile, Wheat and Barley emerge as net shock transmitters in this Quantile. Furthermore, Soybean continually manifests itself as the primary transmitter of spillovers in the studied commodity markets, despite a downward trend in its connectedness. In stark contrast, Soybean Oil and Soybean Meal have displayed an increasing trend in net connectedness. Lastly, while Sunflower's susceptibility to external shocks has grown compared to the middle Quantile, the trend indicates a decrease in its impact from the year 2020 onwards.

### 3.7 Upper Quantile (Q 0.9)

#### 3.7.1 Total connectedness index (TCI) (Q 0.9)

**Chart 10:**
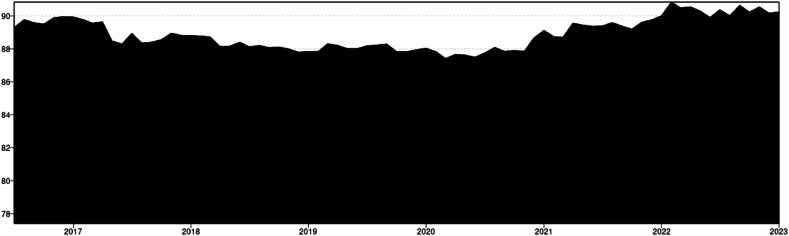
Total connected index (TCI) (Q 0.9).

Analyzing the TCI in upper quantile, specifically the 0.9 quantile or the 90th percentile, provides insights into the behavior of the dependent variable in the upper segment of its distribution. The 0.9 quantile helps in understanding the connectedness of the variables under more favorable or more extreme upper-end conditions. This could include scenarios like market booms, high demand, or high prices. In the context of our research on agricultural and energy commodities, the 0.9 quantile TCI allows us to understand the dynamics of these commodities during periods of significant market booms or upturns. For example, examine how a substantial increase in natural gas prices influences the prices of fertilizers or agricultural commodities in the upper end of their price distribution.

chart 10 illustrates the total connectedness among different commodities for the upper Quantile, currently at 87 %. This relatively high level of connectedness, although slightly lower than the 91 % observed in the lower Quantile, signifies an intense interplay among various commodities. Contrary to expectations, the overall trend demonstrates stable connectedness throughout the COVID-19 pandemic, this is in line with [38] Q 0.9 results. However, an escalation was seen during the intensification of the Ukrainian-Russian conflict, leading to a peak in the connectedness. The conflict caused a major shift in the supply and price of key commodity markets. This change has been one of the most significant since the 2008 financial crisis, indicating a heightened level of connectedness among these commodities and global financial markets [24]**.** The upper quantile exhibits a propensity to absorb positive shocks. This suggests that during the period of conflict, the disturbances in prices were predominantly positive, leading to inflationary pressure on prices.

Timeline & Key events.

1. 2017–2018 (Trade War Onset): Early stages of the U.S.-China trade war. The market disruptions due to tariffs likely led to interconnectedness shifts as supply chains adapted to the new trade environment. However, the TCI remained relatively stable, indicating a consistent level of connectedness during this period or lag effect (200 days rolling windows).
2. 2018–2019 (Trade War Escalation): Escalation of trade tensions and tariffs, as tariffs continued to increase, global commodity markets experienced volatility. The TCI showed a slight increase in connectedness, indicating that commodities became more intertwined, likely as markets reacted to changes in trade policies.
3. 2020 (COVID-19 Pandemic): Despite the global economic turmoil, the TCI remained relatively stable. This aligns with findings from similar research [38], suggesting that during market downturns, the interconnectedness may not increase significantly or a lag effect.
4. 2021 (Post-Pandemic Recovery): Recovery efforts and supply chain issues. The TCI slightly fluctuated but maintained a high level of connectedness, reflecting the impact of heightened demand and ongoing supply chain disruptions in various markets.
5. 2022 (Russia-Ukraine Conflict): The TCI showed a peak during this period, indicating heightened interconnectedness due to supply disruptions and increased prices for key commodities like energy, fertilizers, and agricultural products. The conflict's impact caused one of the most significant market shifts since the 2008 financial crisis, driving inflationary pressures and positive price shocks.

The TCI (Q 0.9) chart highlights that the connectedness between commodities generally remained high and stable, reflecting the interconnected nature of these markets. Significant peaks during the Russia-Ukraine conflict underline the substantial market changes and inflationary pressures that followed, suggesting that during periods of conflict and market booms, commodity markets are highly interdependent.

#### 3.7.3 Total connectedness index upper quantile (Q 0.9)

Table 4, which outlines the TCI in the upper quantile (Q 0.9), reflects how interconnected various commodities are, especially under conditions of market booms, high prices, or high demand. This level of analysis helps in understanding which commodities influence others the most and the intensity of their impact in the commodity market ecosystem. The TCI value is 87 %, indicating that commodities have a significant spillover effect on each other, revealing high levels of interconnectedness during favorable market conditions.

**Table 4:** Total connectedness index (TCI) upper Quantile (Q 0.9).

	Coil	Nga	Bar	SM	Soil	Soy	Sunfl	Wh	USU	USN	FROM
Coil	22.28	7.83	8.22	8.89	11.56	9.91	5.68	7.67	8.81	9.16	77.72
Nga	9.13	19.96	8.65	9.78	9.15	9.31	7.11	8.69	9.82	8.4	80.04
Bar	8.46	6	19.16	12.21	11.18	12.88	6.44	11.12	6.38	6.15	80.84
SM	7.14	6.64	10.37	18.43	11.29	16.66	5.74	11.42	6.12	6.19	81.57
Soil	9.31	6.16	9.5	10.94	18.5	14.32	6.19	10.37	7.62	7.1	81.5
Soy	7.31	5.76	10.66	15.97	13.94	17.87	5.34	11.2	6.15	5.8	82.13
Sunfl	7.67	5.86	8.44	8.49	10.25	8.58	28.66	7.05	5.97	9.03	71.34
Wh	6.92	7.27	10.4	12.47	12.26	12.99	4.19	19.53	6.93	7.05	80.47
USU	8.75	8.38	9.08	9.09	9.88	9.7	6.39	8.9	19.68	10.15	80.32
USN	8.68	7.38	8.06	10.28	11.45	11.25	6.29	8.89	11.7	16.02	83.98
TO	73.38	61.28	83.37	98.11	100.96	105.6	53.37	85.3	69.51	69.04	799.9
Inc.Own	95.65	81.24	102.53	116.54	119.46	123.48	82.02	104.83	89.19	85.06	TCI = 87 %
NET	−4.35	−18.76	2.53	16.54	19.46	23.48	−17.98	4.83	−10.81	−14.94	
NPT	3	0	4	7	8	9	2	6	3	3	

Soybean remains the most influential commodity in the upper quantile with a total spillover of 105.6 % and a net transmission of 23.48 %. Its influence permeates through the market, significantly impacting other commodities, particularly soybean oil (19.46 %) and soybean meal (16.54 %). The importance of soybeans in global food supply chains and biodiesel production is a major factor in its significant influence. These findings approximate to Ref. [38] results and are in line with [60] results. Soybean maintains its influential position across all quantiles examined. This consistency highlights its importance in both lower and higher market conditions, affirming the critical role it plays in global agriculture and energy sectors.

Soybean oil holds the second position with a total spillover of 100.96 % and a net transmission of 19.46 %. This shows that soybean oil's influence is also substantial, impacting not only other vegetable oils (Sunflower 13.94 %) and the soybean complex (Soybean 13.94 %, Soymeal 18.43 %) but also the broader agricultural market due to its role in the food and energy sectors (Barley 11.18 %, Wheat 12.26 %, Natural Gas 9.15 %, Crude Oil 11.56 %, USU 9 % and USN 11.45 %). Its strong influence remains consistent across quantiles, further highlighting its importance. The soybean oil industry's ties to both food processing and biodiesel production contribute to its significant impact.

Soybean meal ranks highly with a spillover of 98.11 % and a net positive balance of 16.54 %. This illustrates its significant impact on animal feed and agricultural markets. The livestock industry's reliance on soybean meal as a protein source underscores its importance. Soybean meal's influence surpasses that of barley and Wheat (NET 2.53 % & 4.86 %), particularly in the upper quantile, showcasing its critical role in higher market conditions due to its extensive use in livestock feed.

Wheat and barley are notable in the secondary sphere of influence, with wheat having a spillover of 85.3 % and barley at 83.37 %. The net transmission rates are 4.83 % for wheat and 2.53 % for barley. These cereals significantly impact the food sector and are key to food security globally. Their consistent position in the secondary sphere across all quantiles demonstrates their steady influence on the global market. Wheat and barley are staples that retain their importance regardless of market conditions.

With a total spillover of 73.38 % and net transmission of −4.35 %, crude oil remains a significant player in the commodity market. Despite being a net receiver at this quantile, it transitions to a net transmitter at lower quantiles, showing the versatility of its influence depending on market conditions. Total spillover for Sunflower oil is at 53.37 % and net transmission at −18 %. Its relatively lower impact compared to soybean oil reflects its smaller share in the global edible oil market.

Phosphate Fertilizer (USN) and Urea (USU) with spillovers of 69 % and 69.51 %, respectively, and negative net transmissions (−14.94 % and −10.81 % respectively), these fertilizers are net receivers. Their dependency on energy markets for production costs ties their influence closely to commodities like crude oil (9.16%v8.68 % and 8.81%v8.75 % respectively) and natural gas (8.4%v7.38 % and 9.82%v8.38 %, respectively).

Natural Gas (Nga**)** with a total spillover of 61.28 % and net transmission of −18.76 %, natural gas is a significant energy commodity but remains a net receiver in this quantile. The gas dispute with Russia has notably increased market volatility, causing it to receive more shocks rather than transmit them [63].

The soy complex, comprising soybeans, soybean oil, and soybean meal, shows significant influence across the quantiles. Their combined impact on global agriculture and energy markets is crucial due to their roles in food supply chains and biodiesel production. Wheat and barley maintain a stable secondary sphere of influence across quantiles, highlighting their critical roles as global staple crops. Their influence remains relatively consistent, reflecting their importance in global food security. The tertiary sphere, which includes commodities like crude oil, sunflower oil, natural gas, and fertilizers, primarily acts as a net receiver of positive shocks. However, their influence fluctuates significantly across quantiles. For instance, crude oil transitions from a net transmitter in lower quantiles to a net receiver in higher quantiles, reflecting its changing dynamics in different market conditions. The significant volatility in the natural gas market, particularly due to geopolitical issues like the Russia-Ukraine conflict, has had ripple effects on the fertilizer market, affecting urea and phosphate prices. This reinforces the interconnectedness between energy markets and agricultural inputs.

In the upper quantile, commodities demonstrate varying degrees of interconnectedness, heavily influenced by their respective roles in the global market. The soybean complex retains its dominant influence, while staple grains like wheat and barley maintain steady secondary influence. The tertiary sphere, including crude oil and fertilizers, reflects the intricate relationships between energy markets and agricultural inputs. Understanding these interconnected relationships can help market participants navigate the complex dynamics of global commodity markets, especially in favorable or extreme conditions.

#### 3.7.5 Network connectedness (NC) upper quantile (Q 0.9)

**Chart 11:**
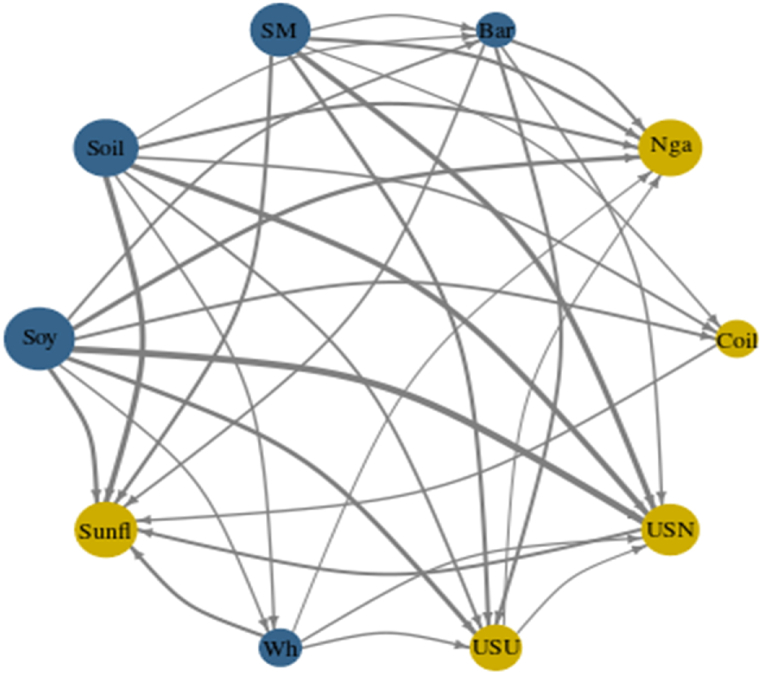
Network connectedness (NC) (Q 0.9).

Chart 11 provides a visual representation of the Network Plot for the 0.9 quantile, highlighting the complex links between various agricultural commodities within this bracket. Once more, for these Quantiles and throughout all of them, Soybean and Soybean products hold the position of leaders in the transmission of price shocks, affecting other markets. The roles of Barley and Wheat as net transmitters resurface in this Quantile, as well as in the lower Quantile, in line with [47] although this trend is not observed in the middle Quantile. Crude Oil reverts to being a net receiver, as in the middle Quantile, and only remains a net transmitter in the lower Quantile. There is a noted lack of interconnection between Crude Oil, Natural Gas, and Phosphate Fertilizer. In contrast with [64] investigation that evaluated how volatility in crude oil and natural gas prices affects fertilizer price variations. It was found that changes in oil and natural gas prices increased fertilizer prices after a crisis period, indicating a significant effect of energy prices on fertilizer prices. As previously described, Sunflower oil appears to be considerably impacted by most commodities. Finally, a spillover effect is evident between Natural Gas and Urea, which seems to flow from Natural Gas to Urea.

#### 3.7.7 Net pairwise directional connectedness (NPDC) upper quantile

**Chart 12:**
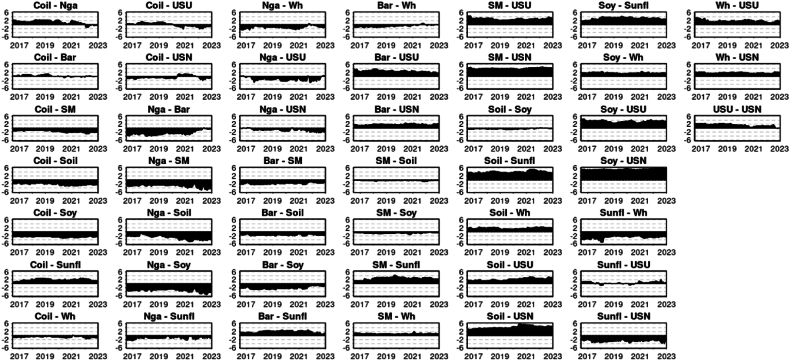
Net pairwise directional connectedness (NPDC) upper Quantile (Q 0.9).

Chart 12 in detail provides a comprehensive understanding of the interconnectedness between commodity pairs under extreme upper-market conditions. This chart captures the influence between commodities, especially during favorable market conditions like market booms and high prices. By understanding each pair's relationship, we can discern the broader market dynamics in the agricultural and energy sectors.

Around 2022, natural gas emerged as a significant net transmitter compared to crude oil, a shift influenced by geopolitical tensions. The Russia-Ukraine conflict and the subsequent dispute between Russia and Europe severely disrupted the natural gas market. Europe's dependency on Russian gas led to significant supply chain disruptions when Russia reduced or halted gas supplies, causing a surge in natural gas prices [65]. As a result, natural gas became a major influencer in the commodity market, impacting the prices of fertilizers and agricultural commodities that rely on it for production. Crude oil, while still influential, saw its relative impact diminish in favor of natural gas during this period [66]. proposed this phenomenon, highlighting that supply shocks can induce deviations from long run equilibrium.

The relationship between crude oil and urea also shows a significant shift. By the end of 2020, urea emerged as a more significant net transmitter than crude oil. This could be linked to the gas dispute between Europe and Russia during the Ukraine-Russia war. The influence of urea grew in the market, reflecting the increasing cost pressures in the fertilizer industry [67,68]. As agricultural production relies heavily on fertilizers like urea, any increase in fertilizer prices directly impacts agricultural costs, creating ripple effects throughout the food supply chain.

The connection between crude oil and phosphate fertilizers reveals an oscillating relationship in net transmission. These fluctuations highlight the delicate balance between energy prices and fertilizer production [69]. Phosphate fertilizers depend on energy inputs, and shifts in global energy markets affect their production and pricing. The interplay between crude oil and phosphate fertilizers demonstrates how global market volatility can influence these commodities' roles as either transmitters or receivers.

Moving to the agricultural sector, soybeans consistently operate as a net transmitter, particularly affecting soybean oil. This strong relationship is due to soybeans being the primary input for soybean oil production, and it has already been described by Ref. [48]. The stability of this relationship underscores the fundamental nature of their connection. Soybeans' influence on soybean oil remains consistent regardless of market conditions, reflecting their integral role in the global food supply chain. Even during favorable market conditions (0.9 Q), where demand for both soybeans and soybean oil remains high, soybeans continue to exert a significant impact in contrast with [48].

In examining the soybean-barley relationship, soybeans consistently emerge as net transmitters. The demand for soybeans, driven by their extensive use in animal feed and biodiesel production, impacts other grains like barley. This interconnectedness is maintained throughout the entire study period, reflecting the consistent global demand for soybeans and their effect on other grains. The agricultural sector's reliance on soybeans ensures their influence extends to related markets like barley, particularly in periods of high demand and favorable prices. Previous empirical evidence neither supports nor refutes this finding. However, the research conducted by Ref. [70] suggests a positive correlation between double-cropping systems involving soybeans and barley, indicating an interconnected relationship between the two commodities. Contrary to the lower Quantile, there is no fluctuation in the net pairwise connectedness sign between Barley and Soybean over the entire period studied. Instead, Soybean consistently operates as a net transmitter, exerting influence on Barley. For the upper Quantile, the increase in connectedness is not as clear as it is in the lower Quantile, nor as it was demonstrated in previous empirical research by Ref. [47].

Wheat and soybeans also share a stable relationship in the upper quantile. Unlike the lower quantile, there is no significant shift in causality in this upper quantile, indicating a consistent relationship between these staple crops. Their stable relationship is influenced by their roles as global staples, with steady demand across market conditions. The significance of both wheat and soybeans in ensuring global food security cannot be overstated. Their mutual importance is evident through the symbiotic relationship fostered by the double-cropping system and their intertwined role in animal feed production [71,72].

Crude oil's relationship with other commodities, like sunflower oil and barley, also provides insights into the broader market dynamics. The influence of crude oil on these commodities (Barley [61], Sunflower [73]) is indicative of the integral role energy prices play in agricultural production. As energy prices fluctuate, so do the production and transportation costs for agricultural commodities, affecting their supply and pricing. This relationship remains consistent, demonstrating how changes in energy markets influence agricultural commodities [73]. assessed the influence of crude oil futures on sunflower seed futures in Hungary. The study highlighted a significant correlation between crude oil and sunflower seed prices, emphasizing the impact of crude oil on the sunflower oil market. The findings align with [73] conclusions until 2020, after which the relationship becomes less distinct and more variable.

Barley and wheat show a close relationship, with barley often acting as a net receiver to wheat. This interconnectedness is influenced by their roles as staple grains in the global food supply chain. Both commodities are subject to similar market pressures [74], including trade policies [75], weather events [76], and global demand shifts. Their stable relationship across market conditions demonstrates the importance of staple grains in global food security.

Soybean meal's influence also stands out in the upper quantile, reflecting its critical role in the livestock feed industry [77]. The relationship between soybean meal and other commodities, like barley and wheat, is influenced by the interconnectedness of the feed industry. The consistent demand for soybean meal, driven by its high protein content, ensures its influence extends across the agricultural sector. Its connection to soybeans and other grains reflects the integrated nature of the feed and food supply chains.

Soybeans and their derivatives, such as soybean oil and soybean meal, consistently influence other commodities due to their critical roles in global food supply chains and biofuel production. The energy sector, particularly natural gas and crude oil, also plays a significant role, influencing agricultural commodities through their impact on production costs. The chart reveals how market dynamics, influenced by geopolitical events, energy prices, and global demand shifts, create intricate relationships between commodities, reflecting the complex interplay between agriculture and energy in the global market.

#### 3.7.9 Net total directional connectedness (NTDC) upper quantile (Q 0.9)

**Chart 13:**
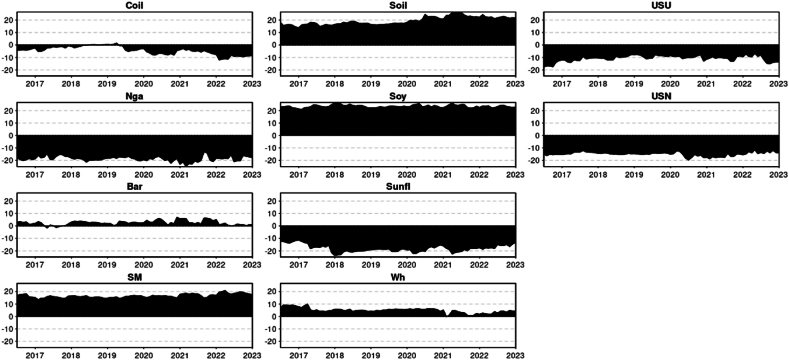
Net total directional connectedness (NTDC) upper Quantile (Q 0.9).

chart 13 displays the net total directional connectedness (0.9 Q) for selected commodities from 2017 to 2023, Crude oil predominantly remains a net receiver of market volatility throughout the period, except for brief intervals in late 2018 and mid-2019 when it acts as a net transmitter. This implies that crude oil often reacts to changes in other commodities, with limited instances of influencing others significantly. This finding reveals a negative correlation between crude oil and agricultural commodities (soybean and its derivatives, barley, and wheat) (0.9 Q). This correlation aligns with the observations made by Ref. [18], which highlighted similar dynamics. Furthermore, the presence of this negative correlation suggests a potential for hedging opportunities. Soybeans consistently exhibit characteristics of a net transmitter, affecting other commodities throughout the entire period. Its role in global agriculture and susceptibility to supply-demand changes ensure that its price fluctuations significantly influence interconnected markets in line with [38].

Following a similar pattern to soybeans, soybean oil also remains a net transmitter. This reflects the considerable influence of soybean oil in food and biofuel markets, where its price shifts create substantial spillover effects [78]. have found significant spillover effects from soybean oil prices to related markets, indicating its role as a transmitter of market information. For instance, correlations between soybean oil and other vegetable oils, such as palm oil, highlight the interconnectedness of these markets and the transmission of price changes. Soybean meal mirrors soybeans in consistently acting as a net transmitter, emphasizing its significance in global markets. Its influence primarily stems from its crucial role as animal feed [78]. Wheat typically functions as a net transmitter, though its level of influence is significantly lower than that of the soybean complex. Its connectedness varies moderately, which corresponds to changes in supply and demand. Building upon the findings of [79], which proposed that the transmission of volatility from the US wheat futures market to China requires more time compared to soybean futures, implying a diminished level of spillover from wheat to other commodities.

Natural gas demonstrates high volatility, often absorbing external shocks from other commodities (Net receiver). This volatility reflects the inherent instability in the global natural gas market. Natural gas prices exhibit high volatility compared to other energy commodities, primarily due to external shocks that arise from fluctuations in supply and demand, geopolitical events [80,81]. Sunflower oil exhibits a similar pattern to natural gas, being highly susceptible to external shocks and prone to market instability. Its connectedness with other markets underlines its vulnerability to broader commodity market trends. For instance Ukraine's sunflower oil market has experienced growth before hostilities and export capacity expansion, but hostilities have negatively impacted the market due to supply chain disruptions and volatile prices [82]. Barley consistently shows characteristics of a net transmitter, though its influence is more subdued compared to soybeans. This suggests that barley's impact on market interconnectedness, while present, is less pronounced.

Fertilizer prices, specifically Urea and DAP are reflected in the chart as USU and USN, respectively. These fertilizer markets influenced mostly net spillover receivers, present varying levels of connectedness, signifying their responses to changes in supply and demand exogenous shocks and other commodities spillovers [55,69,83].

Overall, the chart demonstrates the complex web of interconnectedness among various commodities, with soybeans and their derivatives prominently impacting global price volatility. Conversely, crude oil, natural gas, and sunflower oil are generally more affected by external market factors.

### 3.9 TCI over various quantiles

**Chart 14:**
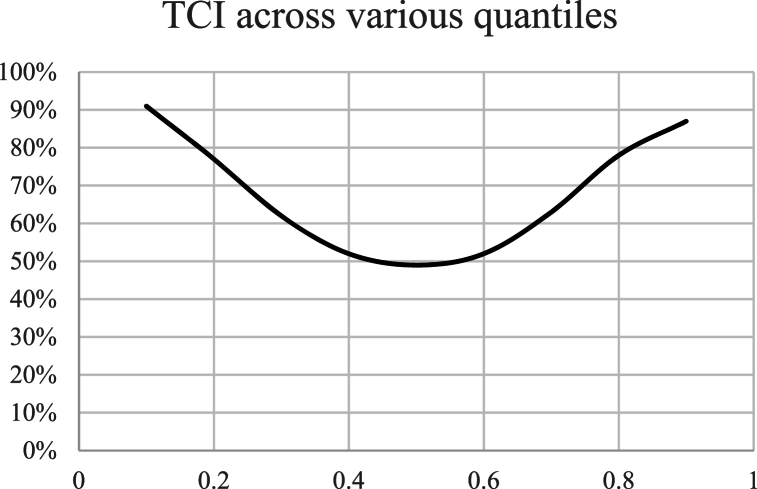
Tci over various quantiles.

Chart 14 represents how connectedness varies across different market conditions, specifically by examining the 0.1 (lower), 0.5 (median), and 0.9 (upper) quantiles. The TCI indicates how interconnected different commodities are, providing insights into their influence on each other across various market conditions. The chart shows that connectedness is highest in the extreme quantiles (0.1 and 0.9), and it gradually decreases towards the median quantile. In simpler terms, this suggests that commodity markets exhibit greater interconnectedness during extreme market conditions, whether in downturns or booms, compared to average conditions.

This pattern indicates a significant degree of similarity in market behavior during periods of high volatility, whether it is a negative shock (such as a downturn or market crash) or a positive shock (like a market boom or bull run). Both the upper and lower quantiles exhibit relatively similar connectedness values, reflecting the absence of asymmetric price transmission among the commodities studied. This implies that when prices change significantly in one direction, it tends to uniformly affect the prices across all commodities.

An example of this uniformity can be seen in the peaks and falls of the TCI across the quantiles. During periods of significant market events, such as the COVID-19 pandemic or the Russia-Ukraine conflict, the commodities exhibit increased interconnectedness. For instance, the global supply chain disruptions during the pandemic impacted energy and agricultural commodities alike, leading to widespread increases in connectedness. Similarly, the Russia-Ukraine conflict created spikes in interconnectedness, particularly in the agricultural and energy sectors.

The median quantile, representing more stable market conditions, shows a relatively lower TCI, indicating reduced interconnectedness among commodities. This reflects a more stable and predictable market environment, where commodity prices are less prone to extreme spillover effects. This pattern highlights the uniform nature of price changes across commodities, suggesting that market disruptions, regardless of their direction, tend to have a broadly similar impact on interconnectedness among commodities.

### 3.10 Granger causality test

Table 5 presents all pairwise causal relationship among the time series in granger causal sense (g-causality). This approach Granger causality is a statistical concept used to determine whether one time series can predict another time series. It is important to note that the term “causality” in this context does not imply a cause-and-effect relationship in the traditional sense; instead, it focuses on the predictive capacity of one series over another. Thus, it cannot be directly compared with the connectedness index approach. However, it remains valuable for contrasting these results with those of the connectedness analysis and complementing the findings. The first notable observation is the almost absence of Granger-causal relationships within the soybean complex. Historically, this crop and its derivatives have exhibited significant influence over most commodities. According to this methodology, these commodities may not serve as reliable predictors for others. While soybean has demonstrated an influence on soybean oil (Soil), leading to a Granger-causal relationship with the latter, this relationship is also confirmed by the connectedness approach. However, in lower Quantiles, the relationship can shift, revealing a bidirectional causality.

**Table 5:**
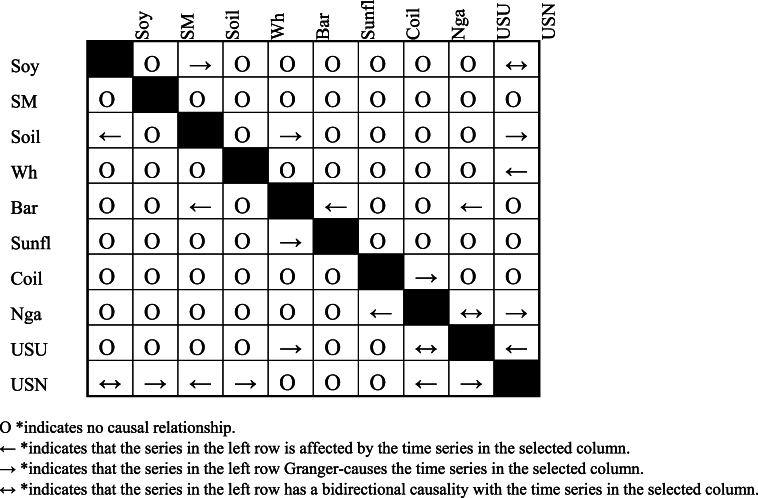
Granger Causality Matrix.

Soymeal (SM) stands out as the only commodity in our sample that does not display a causal relationship with other commodities. This absence could be due to a lack of statistical power in the Granger test, especially considering that soybean meal is derived from soybeans. Intuitively, it should possess a causal relationship with its primary source. Argentina as the leading exporter of soybean meal, commanding a substantial 33 % share of the global market, this country plays a pivotal role in shaping international trade dynamics for this commodity. The Argentine government's interventions in the soybean sector specifically through export tariffs and the manipulation of the currency exchange market can create significant distortions in the price formation mechanisms of soybean meal [31]. Such state-led actions can fundamentally reconfigure the market landscape, precipitating shifts in the economic structure that are not readily discernible through conventional econometric models, including the Granger causality analysis.

These policy instruments wield the power to mold market expectations and incite speculative behaviour among traders and other market participants. As agents within the market strive to pre-emptively respond to anticipated policy manoeuvres, (Change of government, tariff decrease or increase, flexibilization of the currency market) resulting speculative activity can induce price movements that are decoupled from underlying market fundamentals, thereby fading genuine causal linkages or cointegration relationships. Furthermore, the element of policy-induced uncertainty injects a heightened degree of volatility into the market. This volatility does not merely complicate the task of detecting stable time series relationships it calls for a more nuanced analytical framework that can accommodate the complex interplay of policy interventions, market responses, and the resultant economic ramifications.

Soybean oil is Granger-caused by soybean (SOY) and in turn Granger-causes DAP (USN) and barley (Bar). This relationship is corroborated in the pairwise directional connectedness (Q.1 & Q.9). However, other causal relationships evident in the Connectedness approach are not present in the Granger test. This discrepancy appears for the majority of the studied commodities, hinting at a potential limitation in the statistical power of the Granger approach. Similarly, wheat does not exhibit any causality with other crops or energy commodities, being solely influenced by phosphate fertilizer (USN). This is counterintuitive, especially considering that the primary fertilizer for wheat production is urea, yet the observed causal relationship is only with DAP (USN).

Barley (Bar) emerges as the most Granger-caused commodity in the matrix, influenced by soybean oil (Soy), sunflower oil (Soil), and urea (USU). The causal relationship with urea is intuitive from a cost perspective, given that urea is a primary input in barley production. However, the pairwise directional connectedness suggests a bidirectional causality, with barley often driving the price spillover. As previously noted, sunflower oil only Granger-causes barley and does not manifest other causal relationships. Yet, the connectedness approach reveals that the sunflower oil market is highly unstable and significantly impacted by other commodities, positioning it as one of the top net receivers.

Crude oil (Coil) exhibited only one causal relationship, Granger-causing natural gas. While this relationship may initially seem intuitive, since the price of natural gas (Nga) in the European market closely linked to the price of crude oil, given the present significance of conventional long-term contracts that are indexed to the cost of petroleum products [84] The intricate relationship between crude oil and natural gas prices has been a focal point of various empirical studies. Through the application of the NARDL model, research highlights that crude oil prices exert influence on gasoline and natural gas prices, although this influence is neither direct nor linear. Instead, it is characterized by its asymmetric and nonlinear nature, leading to diverse price transmission mechanisms that hold substantial implications for policy [85] Furthermore, the role of global economic trends and dynamics cannot be overlooked [86]. suggest that the global economic milieu, coupled with fluctuations in international crude oil valuations, critically shapes the long-term relationship of regional natural gas import prices. This reiterates the intricate weave of global economies and their impact on energy markets. However, when delving deeper into the interconnections of these markets, the researchers suggest that the primary factors influencing their interactions are demand-side shocks. Interestingly, these shocks have a more pronounced effect than any shifts or alterations in the crude oil supply [87]. Empirical evidence by Ref. [66] indicates that this nexus is mediated through the competition at the margin between natural gas and residual fuel oil. This nuanced understanding suggests that market dynamics are shaped by a multitude of competing forces, each with its own set of influences and implications.

The absence of other causal relationships of crude oil (Coil) with other commodities contradicts the connectedness approach, which indicates that crude oil actively influences other commodities. This discrepancy also contrasts with previous empirical findings [32,53,55]; however is in line with [88,89]. The bidirectional causal relation between natural gas (Nga) and urea (USU) appears logical. Yet, the pairwise directional connectedness primarily shows that its natural gas driving the shock transmission, with occasional shifts in causality during the studied period. Lastly, urea (USU) is Granger-caused by USN (DAP). The connectedness approach indicates this only occurs in the middle Quantile, with a notable causality shift in 2021. However, in extreme Quantiles, urea dominates over USN. This results are in contrast with by Ref. [56] that found that the price of urea Granger causes all other fertilizer prices, including DAP.

Interestingly, USN emerges as the commodity with the most causal relationships, Granger-causing soybean (Soy), soymeal (SM), wheat (Wh), urea (USU), and even itself, while being influenced by natural gas (Nga), soybean oil (Soil), and natural gas (Nga). The connectedness approach indicates that USN is a net receiver, strongly connected with most commodities. However, its net directional connectedness balance remains consistently negative, revealing a commodity highly susceptible to shocks across various markets.

Finally, it is imperative to acknowledge the bidirectional causality observed among certain commodities, particularly between Soybean (Soy) and DAP (USN). Notably, Soybean and DAP exhibit a bidirectional causal relationship. This phenomenon can be attributed to the interdependence of these commodities, where DAP (USN), as a key input in soybean cultivation, influences and is influenced by soybean production dynamics. However, when examining the net pairwise directional connectedness across different market conditions,specifically at the median (Q0.5), lower (Q0.1), and higher quantiles (Q0.9) a distinct pattern emerges. The connectedness analysis reveals a consistent spillover effect from Soybean to USN, indicating that, irrespective of market fluctuations, Soybean predominantly acts as the source of net price spillover. This insight underscores the nuanced interactions between these commodities and their significant impact across various market scenarios. For example [10], suggested that the expansion of soybean production in Brazil, driven by international trade dynamics with China, indirectly impacted the fertilizer market significantly increasing the demand for phosphate fertilizers.

## 4 Conclusion

The research discerned pronounced patterns in the extreme quantiles (0.1 & 0.9) during the studied period, approximately 91 % and 87 % of connectedness. This study discovered that complexity intensifies as it approaches the extreme quantiles (Appendix I). The interconnectedness and dynamics of the soybean market reflect a web of relationships with significant global implications. Analyzing the interplay among various agricultural and energy commodities offers deep insights into market trends and influences, particularly in the context of recent geopolitical events, like the conflict between Ukraine and Russia and the post pandemic era.

Our research findings indicate that soybeans and their derivatives consistently hold a leading position in international markets, acting as the primary source of price spillovers. This was particularly pronounced in the upper quantile (0.9 Q), where the influence of soybeans and soybean meal remained stable, while the risk of spillovers from soybean oil, barley, and wheat intensified, especially during geopolitical conflicts (Ukranian-Russian conflict).

The symmetric nature of spillover effects was evident, with both extreme tails exhibiting high connectedness (0.1 & 0.9) and highlighting the symmetric price transmission. Examining quantiles further clarified the complexities within the interconnectedness of global commodity markets. In the medium quantile (0.5), the Total Connectedness Index (TCI) was lower, indicating a more stable environment with reduced volatility in price spillovers (Normal market conditions). However, the upper quantile revealed a starkly different picture. Here, connectedness surged, reflecting a spike in interdependence across commodities due to market volatility. This phenomenon was heavily influenced by geopolitical crises, like the Russia-Ukraine conflict, which led to significant supply chain disruptions and price increases in both energy and agricultural sectors. The shifting dynamics of price transmission also showcased the impact of energy markets on agricultural commodities. Crude oil consistently acted as a shock transmitter to other energy commodities and fertilizers, particularly natural gas and urea at the lower quantile (0.1 Q). This relationship is reflective of the broader interconnectedness between global energy and agricultural markets.

Interestingly, the interplay between fertilizers and energy commodities demonstrated varying degrees of influence across quantiles. Urea dominated in transmitting shocks to natural gas in the lower quantile, but this relationship reversed later, with natural gas becoming the primary shock transmitter. This suggests a complex interplay influenced by factors like supply chain disruptions and geopolitical tensions that shape the dynamics of agricultural input markets.

Finally, the global soybean market and its interconnectedness with other commodities vividly highlight the cascading effects of major geopolitical events on market dynamics. The pandemic, US-China trade war, and the Russian-Ukrainian conflict each played a significant role in shaping the global commodity markets. The pandemic caused unprecedented disruptions in supply chains, leading to unpredictable shocks, and underscoring the vulnerability of global trade networks. The US-China trade war revealed the resilience of markets, as trade flows adapted to circumvent tariff barriers, yet still left a lasting impact on agricultural prices and supply chains. The Russian-Ukrainian conflict, however, had the most profound spillover effect, markedly intensifying market interconnectedness across energy and agricultural commodities, as seen in the significant spikes in prices and volatility. Together, these events underscore the importance of understanding and managing interconnectedness and risk spillovers in an increasingly complex and unpredictable global market.

### 4.1 Policy implications

In an increasingly globalized market, the interconnectedness of agricultural and energy commodities presents intricate dynamics, as demonstrated by the connectedness approach in this study. This research sheds light on these dynamics, highlighting the systemic importance of these commodities not only in international trade but also in national security and welfare. Recent geopolitical disturbances, such as the Russia-Ukraine conflict, exemplify the interconnected nature of these markets and the far-reaching consequences of such conflicts. Spillover effects can occur across different types of commodities, even without direct relationships between them.

#### 4.1.1 Primary material commodities (soybean, barley, wheat)

• Systematic Reevaluation: Given the pivotal role of primary commodities like Soybean in transmitting market shocks, a systematic reevaluation of their importance is crucial.
• Diversification Strategy: Nations heavily dependent on specific commodities should expand their sourcing strategies and consider alternative options or investigate potential substitutes. This diversification is vital for fostering resilient economies, as exemplified by soybeans, which are a primary protein source for animal feed. The global reliance on soybeans has elevated its prominence among other crops and energy commodities, potentially leading to soybean assuming a role of price leadership and influencing price dynamics across various markets.
• Encouraging the adoption of sustainable practices such as the Round Table on Responsible Soy (RTRS) certification and promoting deforestation-free soybean production are vital strategies to mitigate environmental challenges. These approaches help prevent deforestation, particularly in vulnerable regions like the Amazon and Cerrado, by enforcing strict land-use criteria. Additionally, RTRS certification promotes best agricultural practices, including efficient fertilizer management, which reduces nitrogen pollution and soil degradation. By prioritizing RTRS-certified and deforestation-free soybeans, we can significantly diminish the environmental footprint of soybean cultivation, addressing both contamination and deforestation issues, while fostering a more sustainable and responsible agricultural sector.

#### 4.1.2 Derived products (Soybean Oil, soybean meal and sunflower oil)

• Risk Profile Assessment: The escalating risk profile of derived products like Soybean Oil in geopolitical contexts necessitates targeted strategies to manage these risks. Another significant issue is the pronounced volatility in the sunflower oil market, which is a net receiver of spillover. This instability is heavily influenced by fluctuations in the majority of other commodity markets.

#### 4.1.3 Substitutes

• Encouraging research into substitutes for primary commodities is essential, particularly given the context of market volatility and supply chain disruptions. Similarly, developing alternatives to soy meal for animal feed is crucial for reducing global reliance on soybeans and mitigating the environmental impact of soybean cultivation.
• Policy Support: Implement policies that support the adoption and scalability of these substitutes, ensuring they are economically viable and accessible.

#### 4.1.4 Fertilizers commodities (DAP & urea)

• Ensuring Availability: Policymakers must ensure the consistent availability of essential fertilizers like DAP & Urea through domestic production incentives, strategic trade partnerships, or subsidy frameworks.
• Risk Profile Assessment: Agricultural enterprises, farmers, and financial institutions must recognize the interconnections among various agricultural commodities, energy resources, and fertilizers. This is particularly crucial concerning the relationship between natural gas and urea. Price shocks in natural gas directly impact urea prices. Furthermore, urea and DAP (Diammonium Phosphate) are integrally linked with all agricultural crops, with fertilizers often acting as the primary receiver of price spillovers in these markets

Furthermore, the study underscores the need for a paradigm shift in energy policies, advocating substantial investments in renewable energy sources to balance environmental stewardship with strategic imperatives. The role of essential fertilizers in agricultural productivity emphasizes their indispensability in this matrix.

The insights concerning extreme quantile behavior, which often delineate market volatility patterns, could be instrumental in establishing robust early warning systems. Financial institutions and regulatory bodies must consider these interconnectedness insights, refining financial instruments, derivatives, and risk evaluation methodologies.

Lastly, in a world where geopolitical tensions can impact global markets, diplomacy and multilateral collaboration are paramount. Strengthening diplomatic avenues to pre-empt conflicts or mitigate market implications is essential for maintaining market stability and fostering international cooperation.

## Data availability statement

Data will be made available on request.

## CRediT authorship contribution statement

**Gustavo María Barboza Martignone:** Writing – review & editing, Writing – original draft, Validation, Resources, Project administration, Methodology, Investigation, Formal analysis, Data curation, Conceptualization. **Bikramaditya Ghosh:** Visualization, Formal analysis, Data Curation. **Dimitrios Papadas:** Supervision, Project administration, Conceptualization. **Karl Behrendt:** Writing – original draft, Supervision.

## Declaration of generative AI and AI-assisted technologies in the writing process

During the preparation of this work the author(s) used ChatGPT 4 to improve the English. After using this tool/service, the author(s) reviewed and edited the content as needed and take(s) full responsibility for the content of the publication.

## Declaration of competing interest

The authors declare no competing interests.

## Figures and Tables

**Figure:**